# Synchrony of Sylvatic Dengue Isolations: A Multi-Host, Multi-Vector SIR Model of Dengue Virus Transmission in Senegal

**DOI:** 10.1371/journal.pntd.0001928

**Published:** 2012-11-29

**Authors:** Benjamin M. Althouse, Justin Lessler, Amadou A. Sall, Mawlouth Diallo, Kathryn A. Hanley, Douglas M. Watts, Scott C. Weaver, Derek A. T. Cummings

**Affiliations:** 1 Department of Epidemiology, Johns Hopkins Bloomberg School of Public Health, Baltimore, Maryland, United States of America; 2 Institut Pasteur de Dakar, Dakar, Senegal; 3 Department of Biology, New Mexico State University, Las Cruces, New Mexico, United States of America; 4 Office of Research and Sponsored Projects, University of Texas at El Paso, El Paso, Texas, United States of America; 5 Center for Biodefense and Emerging Infectious Diseases and Department of Pathology, University of Texas Medical Branch, Galveston, Texas, United States of America; 6 Department of Microbiology and Immunology, University of Texas Medical Branch, Galveston, Texas, United States of America; University of Oklahoma Health Sciences Center, United States of America

## Abstract

Isolations of sylvatic dengue-2 virus from mosquitoes, humans and non-human primates in Senegal show synchronized multi-annual dynamics over the past 50 years. Host demography has been shown to directly affect the period between epidemics in other pathogen systems, therefore, one might expect unsynchronized multi-annual cycles occurring in hosts with dramatically different birth rates and life spans. However, in Senegal, we observe a single synchronized eight-year cycle across all vector species, suggesting synchronized dynamics in all vertebrate hosts. In the current study, we aim to explore two specific hypotheses: 1) primates with different demographics will experience outbreaks of dengue at different periodicities when observed as isolated systems, and that coupling of these subsystems through mosquito biting will act to synchronize incidence; and 2) the eight-year periodicity of isolations observed across multiple primate species is the result of long-term cycling in population immunity in the host populations. To test these hypotheses, we develop a multi-host, multi-vector Susceptible, Infected, Removed (SIR) model to explore the effects of coupling multiple host-vector systems of dengue virus transmission through cross-species biting rates. We find that under small amounts of coupling, incidence in the host species synchronize. Long-period multi-annual dynamics are observed only when prevalence in troughs reaches vanishingly small levels (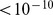), suggesting that these dynamics are inconsistent with sustained transmission in this setting, but are consistent with local dengue virus extinctions followed by reintroductions. Inclusion of a constant introduction of infectious individuals into the system causes the multi-annual periods to shrink, while the effects of coupling remain the same. Inclusion of a stochastic rate of introduction allows for multi-annual periods at a cost of reduced synchrony. Thus, we conclude that the eight-year period separating amplifications of dengue may be explained by cycling in immunity with stochastic introductions.

## Author Summary

Dengue virus has been isolated from mosquitoes, non-human primates and humans in Senegalese jungles for the past 50 years. This sylvatic cycle shows unique transmission dynamics that are unexpected given previous theory and observation: First, the isolations appear to be synchronized across several host and vector species each with different natural histories of infection. Second, the periodicity of the isolations (time between outbreaks) is approximately eight years, much longer than the one or two year period observed in human endemic settings (e.g., Thailand, Brazil). In this paper we develop a multi-host, multi-vector differential equation model to test hypotheses that are potentially consistent with these observations. We find that coupling of separate primate-mosquito pairs through mosquito biting induces synchrony that is robust over a wide range of parameters. We also find that the eight year cycle is not robust to the inclusion of a constant introduction of infection, but is to a stochastic rate of introduction, and thus may be due to cycling of immunity among primates with long-period stochastic introductions. An accurate and thorough understanding of the sylvatic cycle of dengue may allow prediction of epidemics and lessen its impact on humans living in surrounding areas. This knowledge is especially important given the potential for these primate species to act as reservoirs for dengue in post-vaccination scenarios.

## Introduction

Dengue virus occurs in two distinct transmission cycles: transmission among non-human primates (and occasionally among humans) by *Aedes* and other mosquitoes in the forest canopy (the sylvatic cycle) and transmission among humans primarily by *Aedes aegypti* in rural villages and urban communities (the human cycle) [1]. While there is evidence to suggest maintenance of transmission exclusively among non-human primates in Western Africa and Malaysia with occasional spillover to humans, the precise role of particular primates in the sylvatic dengue transmission cycle is unknown [2], [3], [4]. A sylvatic cycle of dengue virus has been documented in Senegal by the detection of dengue-2 antibodies and isolation of sylvatic dengue-2 virus from non-human primate blood [2]. Sylvatic dengue-2 virus has also been isolated from mosquitoes captured in the gallery forest [2]. Though sylvatic and endemic human strains are genetically distinct, they perform similarly in many experimental assays that characterize transmissibility [5], suggesting that the sylvatic strains have a high potential for emergence as human pathogens [6]. Furthermore, several studies have demonstrated that sylvatic dengue strains can cause febrile illness and hemorrhagic syndromes in humans [7], [5], [8], [9], [10] and that infections with sylvatic or human dengue strains are clinically indistinguishable [10], [11].

**Figure 1:**
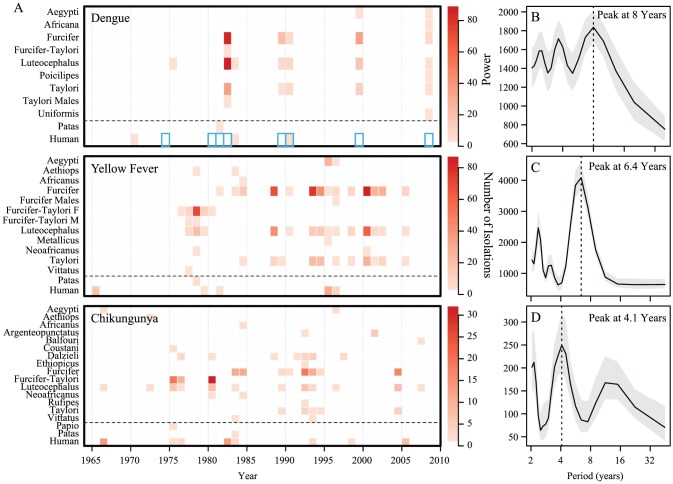
Summary of dengue, yellow fever and chikungunya isolates, 1962–2008. Panel A shows number of dengue, yellow fever and chikungunya virus isolates over time by species. Scales at right indicate number of isolations. Blue boxes on dengue heatmap indicate sylvatic human isolations reported in Diallo et al. (2003). Dashed black lines separate mosquito isolations from primate isolations. Panels B, C and D show the Fourier power spectrum with Daniell smoothers of (3,3) with 95% bootstrap confidence intervals for the aggregated dengue, yellow fever and chikungunya virus isolates, respectively. A detailed description of surveillance methods has been published previously [2].

Routine surveillance for multiple mosquito-borne viruses has been conducted in southeast Senegal for over 50 years by the Institut Pasteur. Surveillance is performed by capturing mosquitoes via-human landing collection in the gallery forest as well as periodic and opportunistic capture of primates (Figure 1, note: Yellow fever and chikungunya isolations are included to show that periods of inactivity in the dengue time series is not due to a lack of collection activities [2]). The data show several patterns: First, there is a dominant eight-year period in the power spectrum of the dengue isolate time series, which is lengthy compared to the commonly observed periodicities in endemic settings of seasonal dynamics often accompanied by a 2–4 year multi-annual cycle. Second, there is strong synchrony of outbreaks: isolations appear to occur in all species during the same year, though most of the isolations are from vectors and fewer have been from vertebrate hosts. Third, the dynamics across dengue, yellow fever and chikungunya appear to differ, with large outbreaks of each occurring at different periodicities and not overlapping.

Host population demographics are known to play important roles in the dynamics of many infectious diseases [12]. Particularly, the rate of recruitment of susceptibles via birthrate is a key determinant of the periodicity of measles, pertussis, and dengue epidemics [13], [14], [15], [16], [17]. It has been hypothesized that the long-period cycle of sylvatic dengue is driven by population turnover and the cycling of herd immunity in non-human primates [1]. For a given primate species, a decrease in the birth rate could elongate the inter-epidemic period. Hence, if transmission in the two different host species is independent, we would expect to see primates with higher birth rates having shorter inter-epidemic periods than primates with lower birth rates, driving the two hosts out of synchrony; however, the observed data suggest synchronized epidemics (Figure 1).

In the current study, we aim to explore two specific hypotheses: 1) primates with different demographics (birth rates) will experience outbreaks of dengue at different periodicities, when observed as isolated systems, and that coupling of these subsystems through mosquito biting will act to synchronize incidence; and 2) the eight-year periodicity of dengue incidence observed across multiple primate species is the result of long-term cycling in population immunity in the host populations. We employ a Susceptible, Infected, Removed (SIR) model to examine the dynamics of dengue transmission in a hypothetical system of coupled non-human primate populations, each with its own mosquito vector. We focus on two features of the dynamics: the period of oscillations in incidence and the correlation of incidence in the multiple host species. To our knowledge, this is the first model of sylvatic dengue and the first to model transmission of dengue among multiple hosts and vectors. This work is exploratory and aimed at characterizing the dynamics of sylvatic dengue in Senegal over a broad range of parameterizations to begin to understand the basic behavior these systems can show and which broad classes of models are consistent with the observed data.

## Methods

### Basic SIR Model Formulation

**Figure 2:**
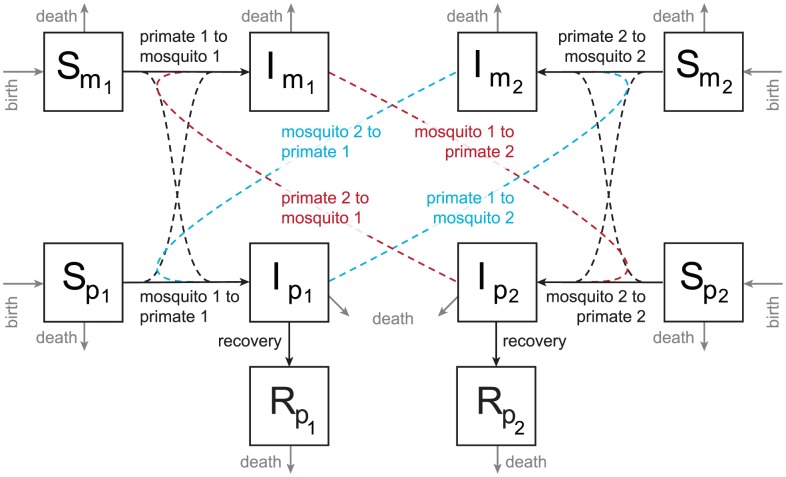
Diagram of SIR model. The model incorporates two primate species and two mosquito species that are coupled through the blue and red cross-biting rates. Each mosquito species is assumed to have a preferred host; these transmissions are represented in black. Each transmission term incorporates two aspects: one, a biting rate between mosquito 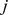 and primate 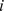 which is symmetric (e.g. “mosquito 2 to primate 1”), as well as a seasonally-varying probability of infection term which is asymmetric for primates and mosquitoes. Primates recover at rate “recovery”. Mosquitoes and primates birth and death rates are represented in grey (labeled “birth” and “death”, respectively). See the text for more detail, and the Supporting Information S1 for model equations.

Our deterministic SIR model extends a framework presented in Keeling and Rohani (2008) [12]. Figure 2 illustrates the two-host, two-vector case. Briefly, mosquitoes and primates are born susceptible to dengue viral infection, and are infected at a rate proportional to the number of bites given or received per day and the probability of infection which we assume is asymmetric for mosquitoes and primates (i.e.: the probability of infection from mosquito to primate is not equal to the probability of infection from primate to mosquito). These transmission probabilities vary seasonally to represent the fluctuation in per bite transmission probability due to seasonally varying processes [18]. After infection, primates recover at a fixed rate and mosquitoes are infected for the remainder of their life. We assume no disease induced mortality in primates [19], [20]. Full model equations are given in the Supporting Information S1 and parameters are defined in Table 1.

**Table 1:** Model parameters.

Parameter	Description	Baseline Value
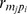	Biting rate/day to primates by mosquito 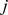 [2], [47]	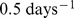
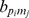	Transmission probability,	0.15
	from primate 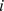 to mosquito 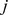	
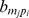	Transmission probability,	0.15
	mosquito 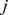 to primate 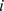 [2], [39], [5]	
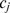	Percent of the magnitude of seasonal variation	between 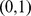 ,
	for mosquito 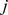	0.05 unless otherwise specified
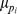	primate birth rate ( = 1/lifespan)	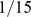 to 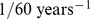
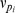	Primate death rate, set equal to primate birth rate	
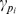	Primate recovery rate [48], [49], [50]	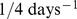
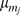	Mosquito 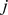 birth rate	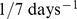
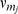	Mosquito death rate, set equal to mosquito birth rate	
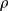	Mosquito rate of transovarial transmission	0
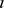	Rate of infectious introduction	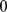 to 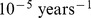

Additional justifications for baseline values are described in subsequent sections.

In this paper, we focus briefly on the one-vector (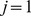), one-primate case (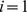), and then on the two-vector (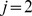), two-primate case (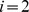). Two-vector, one-primate systems are examined in the Supporting Information S1. We can represent the different biting rates of each mosquito species on all primate species through a matrix, 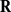:
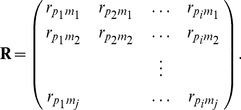
(1)


For each primate-mosquito subsystem, we assume that each primate has a vector species that is source of the largest number of bites that could transmit dengue (we term this the principal vector species). We organize the biting matrix such that the biting rate of the primate species experiencing the largest number of bites is on the diagonal (we term them the *on-diagonal* biting rates) and the primate species experiencing the lower number of bites is off the diagonal (we term them the *off-diagonal* biting rates). These terms are used for notational convenience as 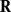 is not required to be square: our model allows different numbers of hosts and vectors with varying degrees of vector preference. Here, to test the hypothesis that coupling of incidence is induced through cross-species biting, we assume that because of spatial segregation of hosts there are host-vector pairs with the vector having one preferred host and one less-preferred secondary host that is bitten much less frequently (i.e., off-diagonal biting rates are less frequent than the on-diagonal biting rate), however we vary this assumption from no cross-biting to equal on- and off-diagonal rates (see Supporting Information S1: Figure S4). Thus, the use of the term “principal vector” is for mathematical convenience as the conclusions drawn do not depend on excluding equal cross-biting cases.

The force of infection of mosquitoes upon primates is density dependent, but the force of infection of primates upon mosquitoes is frequency-dependent. This can be illustrated simply in the one-host, one-vector case by focusing on the rate of change of the infectious compartments for mosquitoes and primates due to infection:
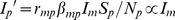
(2)

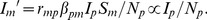
(3)Here 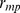 is the biting rate, 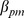 the per-bite infection probability from primate to mosquito, 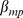 the per-bite infection probability from mosquito to primate, 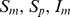 and 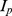 the number of susceptible and infectious mosquitoes and primates, respectively, and 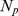 the total number of primates. An increase in the density of infectious mosquitoes directly increases the force of infection for the primate, while an increase in the prevalence (frequency) of infection in the primate population directly increases the force of infection for the mosquito. Conceptually, the number of bites *taken* by a single mosquito is independent of the number of other mosquitoes and primates but the number of bites *received* by a primate increases as the number of mosquitoes increase and decreases as the number of other primates increase. Thus for a primate, an increase in the density of infectious mosquitoes will increase its risk of infection, and an increase in the number of other primates will “dilute” the number of infectious mosquito bites and decrease its risk of infection. For a mosquito, a higher frequency of infection in primates will increase its risk of infection as it becomes more likely to feed upon an infected primate [12], [21].

Finally, we model two forms of the denominator of the force of infection:
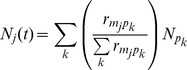
(4)and
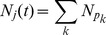
(5)


The first weights the sum of the primate population by the on– and off-diagonal biting rates and the second does not [22], [23]. Each corresponds to an extreme of mosquito behavior: Equation 4 models the situation where the mosquito hones in on hosts with great efficiency, while Equation 5 models the situation where mosquitoes might have innate preferences but be confused by the environmental cues of other species (e.g. carbon dioxide, organic volatile body odors, air movement, heat) used to find preferred hosts, and feed on whatever host it first encounters [24]. We include multiple formulations of this term because there is not sufficient data to rule one pattern of biting out over another and there has not been a consensus in the literature (see Supporting Information S1). The first is examined in the main text, the second in the Supporting Information S1.

### Examining Model Outputs

**Figure 3:**
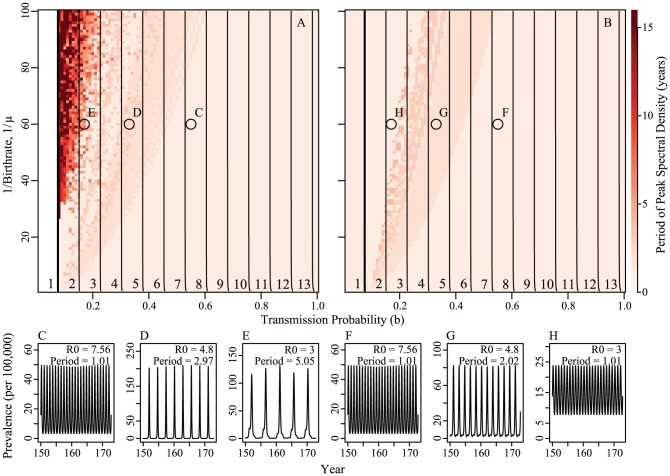
Effect of demographics on model dynamics with and without constant introduction. This figure displays the effects of changing transmission probabilities (x-axis) and 1/primate birth rates (y-axis). Panels A and B are heatmaps of the period of peak Fourier spectral densities in the 1-host, 1-vector systems, with and without 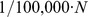 per year rate of infection introduction, respectively. Circles indicate example epidemic time series shown in panels C–H. Contour lines are analytically calculated values of 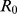 (see Supporting Information S1). Other parameters held fixed: 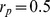, 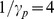, 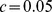, 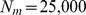, and 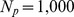.

For each of the model structures and parameterizations, we characterize several aspects of model behavior in order to assess the consistency of behaviors though multiple outputs. Figure 3 shows the output used to characterize the behavior of our models. Panel A shows a heatmap of the period at which maxima in the Fourier spectrum of each simulated series occur, with transmission probability, 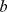, on the x-axis and the life span ( = 1/birthrate, 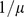) on the y-axis. The contour lines are values of 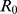, which are derived in the Supporting Information S1. The Fourier spectra were calculated over a period of 50 years after numerically integrating the system for 150 years to eliminate transient behaviors [25]. Log transforms of the state variables were used to minimize numerical error in the integration. Panels C, D and E show example time series with parameter values taken from the position indicated on the heatmap, with the time units being years from the start of integration. Throughout, we hold all parameters fixed except those under investigation. We focus on the dynamics in primates as the mosquito dynamics are nearly identical (see Supporting Information S1: Figure S5).

### Model Parameters: Host Demographics

We vary birth rates in both the single and multi-host/vector systems to determine the effect on the periodicity of dengue prevalence. In the multi-host system, we consider a larger primate with a birth rate of 1/60 years and a smaller primate with a birth rate of 1/15 years. Three primate species from which dengue has detected in Senegal are the African green monkey (*Chlorocebus sabaeus*), the patas monkey (*Erythrocebus patas*) and the baboon (*Papio papio*), which have life-spans of 11, 20 years and 40 years, respectively [26], [27]. Recent age-stratified serosurveys have identified dengue-neutralizing antibodies in 81% of green monkeys, 32% of Patas monkeys and 73% of baboons (Cummings et al., in prep.). The population density of each of the primate species in Senegal is unknown. To examine the effect of relative population sizes on model dynamics we vary the numbers of primates from 1 to 10,000 while keeping the number of mosquitoes fixed at 25,000.

### Model Parameters: Coupling through Vector Feeding on Multiple Species

For the multi-host, multi-vector system, we examine two interacting systems (for example, African green monkey– *Ae. furcifer*, and patas– *Ae. taylori*). It is unclear whether there are strong preferences of the vector for particular hosts, or more likely, vectors feed on available hosts without a preference. The latter may still show large disparities in host biting due to spatial separation of vectors and hosts into particular habitat. Our model formulation can represent coupling of two distinct meta-populations of a single species or *Aedes* feeding on multiple primate species. Here, the off-diagonal biting rates, would represent the rate at which these groups intermix. We explore a broad range of coupling rates.

Quantifying the rates of coupling between the two systems is difficult in practice. Although several studies have shown the anthropophilic feeding habits of *Ae. aegypti*, the bulk of the studies examining multiple-host blood meals in species of *Aedes* do not include non-human primates. This is mostly due to technical difficulties in the differentiation of human and non-human primate blood in mosquitoes [28], and the assumption that there is no involvement of non-human primates in most dengue transmission cycles.

In our model, we can couple the systems by adjusting the off-diagonal biting rates, 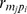, where 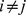. We range the coupling fraction from 0 to 100% of the preferred species biting rate (here taken to be 0.5).

### Model Parameters: Rates of Constant Introduction of Infection

We consider three cases: first, a closed system with no immigration or emigration of infected individuals, second, an open system with a constant introduction of infectious individuals into each non-human primate population, and third an open system with stochastic introductions. It is unknown how isolated populations of non-human primates in Senegal are from other surrounding populations. Sylvatic dengue circulates throughout West Africa in strains genetically distinct from both endemic strains and other (southeast Asian) sylvatic strains [10], [6], [29]. Troupes of patas and baboons, common in Senegal, have been shown to travel up to 12 and 14 kilometers in a day respectively [30], [31], [32]. Groups of green monkeys in Senegal have been shown to occupy broad geographic areas, and routinely interact with individuals far from their home territories [33]. The use of a constant rate of introduction allows us to represent migration of infectious non-human primates into the modeled populations from surrounding populations. In light of the fact that the effective population sizes and migration rates are unknown, we vary population sizes and make qualitative inferences about the dynamics, keying in on behaviors that are robust to assumptions about the total population size. We also examine models with vertical transmission of infection between mosquitoes.

### Model Parameters: Vertical Transmission

Transovarial transmission is often suggested as a hypothesis for sylvatic dengue maintenance [3]. Minimum-infection rates from collections of *Ae. aegypti* larva from dengue–endemic areas range from 0.259/1000 in Rangoon [34] to 28.0/1000 in Chennai, India [35], with estimates from other studies falling towards zero (see [3] pp. 26–9). Additionally, lab evidence has demonstrated seven generations of sustained transovarial transmission of dengue-3 in *Ae. aegypti* (at a large fitness cost to the mosquito [36]), and a modeling study has demonstrated the possibility of overwintering of dengue in mosquitoes, this was, however, in an endemic setting for a single season [37]. We model vertical transmission by allowing a proportion of infected mosquitoes to transmit dengue virus to their offspring.

### Model Parameters: Transmission Probabilities and Seasonality

In Senegal, the main vectors of dengue virus are *Ae. furcifer*, *Ae. taylori*, *Ae. luteocephalus*, *Ae. vittatus* and *Ae. aegypti* [2]. Studies of these vectors differ widely in their estimates of vector competence. While this may be due to differences in study designs (sample sizes, blood meal titer concentration, etc), it suggests large variation in transmission probabilities for *Aedes* mosquitoes, and that they are difficult parameters to accurately estimate (see [38] and [12] p. 137). Here we assume transmission probabilities are seasonally forced 5% a year with a baseline value of 0.15 and mosquitoes deliver an average of 0.5 infectious bites per day [2], [39], [39,5]. In the Supporting Information S1, we include explorations of the biting and transmission rates, differences in the magnitude of seasonal forcing (higher and lower than 5%), seasonality modeled as changes in mosquito birthrates (not transmission probabilities), a 2-vector/1-host system, and an additional formulation of the frequency dependence term.

### Stochastic Model

We developed a stochastic version of the model simulated using a Gillespie stochastic simulation algorithm [40] with the Binomial Tau leap approximation (BTL) [41] to examine the effects of population size on dengue isolation periodicity. BTL was chosen here for efficiency, computational speed and to avoid negative population sizes [41], [42]. Parameters explored were chosen to be identical to those in the main text besides primate and mosquito population sizes which were chosen as a balance between realism and computational efficiency.

## Results

### Models Varying Host Demographics

#### Single host

We begin by characterizing the behavior of the single host, single vector system. Figure 3 indicates that for lower birth rates (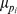 between 1/50 years to 1/100 years) we see multi-annual cycles when transmission probabilities (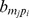 and 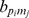) are low. There is little influence of birth rates on 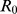 (the slopes of the contour lines are close to zero as birth rate increases).

#### Multi-host

Similar to the single host system, we see multi-annual cycles in the multi-host, multi-vector system when mosquito transmission probabilities are low, host species have low birth and death rates, and there are high numbers of primates relative to mosquitoes.

**Figure 4:**
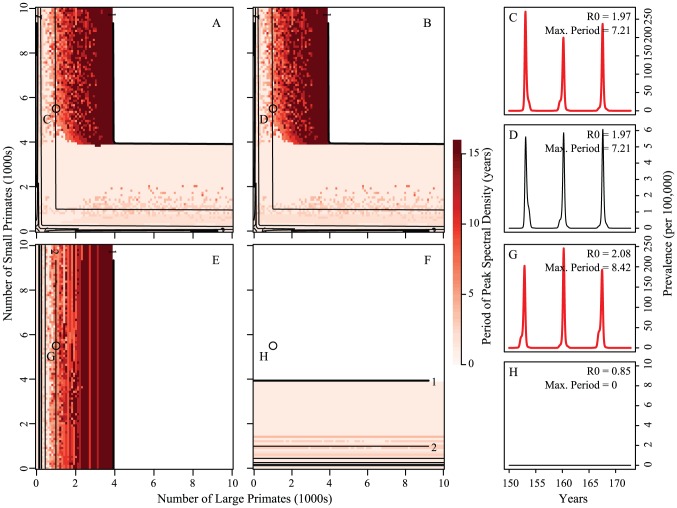
Prevalence in large and small primates in the coupled and uncoupled systems without constant introduction. Panels A and B show results for models with coupling, E and F for uncoupled models. Panel A and E characterize the dynamics of dengue in the large primate species, B and F dengue dynamics in the small primate species. Coupled models (A, B, C and D) are coupled at 1/500th of the on-diagonal biting rates. Panels C, D, G and H show time series for large (C, G) and small primates (D, H) with parameters indicated by the circles in panels A, B, E and F (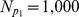 and 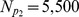). The only parameter difference between panels A–D and panels E–H are the off-diagonal biting rates. Contour lines are analytically calculated values of 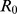 (see Supporting Information S1). The dynamics in the mosquito population are qualitatively identical and can be found in Figure S5. Other parameters are: 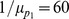, 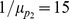, 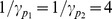, 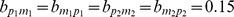, 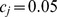, and 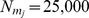, 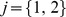.

Figure 4 shows both the effect of relative numbers of primates and mosquitoes, and the effect of coupling on the system. With high numbers of large and small primates relative to mosquitoes, 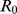 drops to less than one and sustained transmission is impossible (white area in top right corners of Figure 4: Panels A, B, E, F). This is consistent with previous results that infectious mosquito bites are diluted and transmission is reduced in systems with low mosquito-to-primate ratios [43].

When the total number of primates is lower, multi-annual cycles are possible. With small amounts of coupling (1/500th of on-diagonal biting), we find long-period cycles only when the numbers of small primates are higher than the numbers of large primates. In this situation, the value of 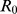 for the small primate (calculated with the number of large primates held fixed) drops below 1 and the inter-epidemic period is driven by the large primate. This is seen in the uncoupled system (Figure 4: Panels E and F). Thus, the region most consistent with the data – regions of long-period, synchronized cycles – has the larger species (with the lower birth rate) exhibiting a higher force of infection (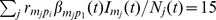) than the smaller species with the higher birth rate (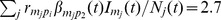). Additionally, when coupled, we see synchrony in epidemics; the dynamics are similar for both large and small primates (Figure 4 : Panels C, D). Coupling the system through vector feeding on multiple species causes the phases of large and small primate epidemics to synchronize. Increasing the coupling to even modest levels causes epidemics to synchronize and that synchrony holds over a broad range of parameters (see Supporting Information S1: Figures S3 and S4).

**Figure 5:**
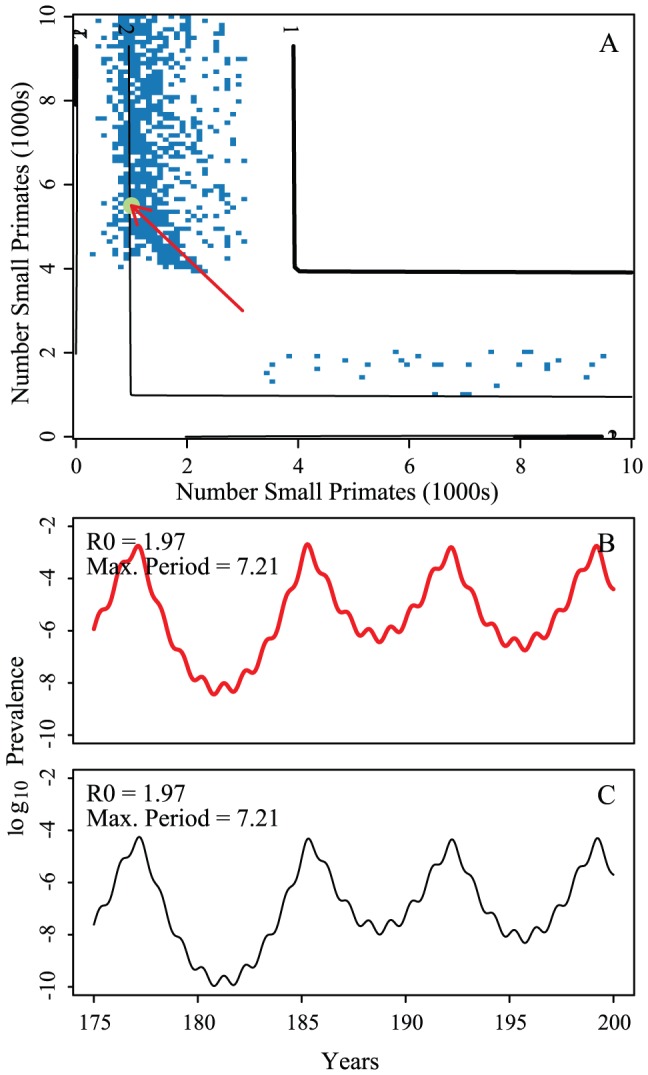
Example time series of long-period isolations. This figure indicates the regions of model parameter space that exhibit multiannual dynamics consistent with the observed periodicity of isolations of dengue in Senegal. The blue dots highlight areas of panel A in Figure 4 where the Fourier spectrum has a maximum between 5 and 12 years. The figure also shows an example time series of long-period, synchronized cycles in large primates (panel B) and small primates (panel C). The arrow and green dot indicate the position in parameter space that was used to generate the time series in panels B and C. Here, 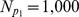 and 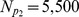 are coupled at 1/500th of the on-diagonal biting rates. Contour lines are analytically calculated values of 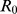 (see Supporting Information S1). The dynamics in the mosquito population are qualitatively identical and can be found in Figure S6. Other parameter values are: 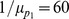, 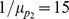, 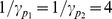, 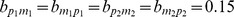, 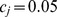, and 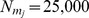, 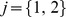.

Even though these long-period regions are found over a fairly large and realistic range of parameters (highlighted in blue in panel A of Figure 5), these regions have vanishingly small prevalence between outbreaks. Prevalence in these troughs reach lows of 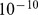 infected primates, which motivates the inclusion of a constant introduction of infected individuals, explored in the next section.

### The Impact of Constant Introduction of Infection and Vertical Transmission

The addition of a constant rate of introduction of infected primates into both populations causes most multi-annual cycles to shorten. Panels B, F, G and H of Figure 3 are identical to panels A, C, D and E, but with a constant introduction rate of 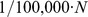 per year. The longest multi-annual period now observed is 4 years. Coupling the two systems still induces synchrony of epidemics. An analogous figure to Figure 4 but with constant introduction is presented in the Supporting Information S1 (Supporting Information S1: Figure S2).

The inclusion of vertical transmission at rates from 0 to 100% of mosquito births does not qualitatively change the impact of coupling. Similarly to the inclusion of a constant rate of infectious introduction, it tends to reduce the length of the multi-annual cycles (results not shown).

### Stochastic Model

**Figure 6:**
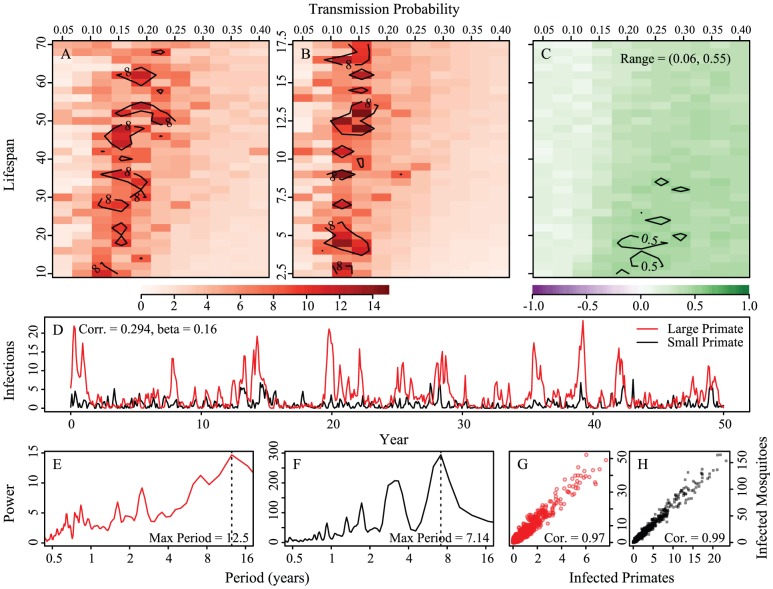
Stochastic formulation of the model. Heatmap of the period of maximum Fourier spectra with corresponding example epidemic time series of prevalence. Panels A, B and C compare transmission probabilities (x-axis) and 1/birth rate (y-axis) for the large primate (panel A) and the small primate (panel B). Birthrates for the small primate are 1/4th of those of the large. Panel A shows periods of oscillations for large primates, B, periods of oscillations for small primates and C the correlation of the mean number of cases in a year (all panels are averaged over 25 runs). D is an example realization of the model with long-periodicity; 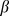 for both hosts and vectors equal to 0.16 and 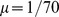 and 1/17.5 for the large and small primates, respectively. Fourier spectra for the large and small primate time series are shown in panels E and F, respectively. Panels G and H are scatterplots of the number of primate infections versus number of mosquito infections for the large and small primates and their corresponding mosquitoes, respectively. We see transmission dynamics in primates and mosquitoes are highly correlated. The coupling is 1/100 of the on-diagonal biting rates; other parameters are: 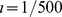, 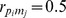, 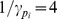, 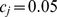, 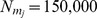, and 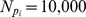, 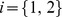, 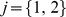.

Figure 6 shows the results of the stochastic formulation. There are long-period cycles even when including a constant rate of introduction equal to 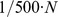. Comparing the stochastic and deterministic formulations of the model, we observe multi-annual dynamics over similar parameter ranges. However, the stochastic version shows 8–10 year cycles with infectious introduction whereas the deterministic model shows shorter periods (Figures 6 and 3). With the chosen population sizes, we do find extinction events of dengue, followed by reintroductions from the relatively constant rate of infectious importation. As expected, we find highly correlated transmission dynamics between the mosquitoes and primates (Figure 6, panels G and H). The stochastic formulation reduces the effect of synchrony. In the stochastic realizations, all parameterizations result in correlation coefficients of the annual aggregate data of less than 0.6 (Figure 6, panel C). However, in accordance with our hypotheses, stochastic realizations without coupling show little to no synchrony at all (see Supporting Information S1: Figure S12). The deterministic models show high correlation (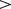
0.9) across a wide range of parameters (see Supporting Information S1: Figure S3). Thus in opposition to deterministic models, stochastic models with coupling and constant introduction show multi-annual dynamics, but with reduced synchrony.

### Robustness of Results to Other Parameters

As presented in the Supporting Information S1, the conclusions drawn above (the small primate determines the periodicity of isolations when its 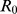 is greater than 1, coupling primate systems induces synchrony and adding a constant introduction of infection causes the length of the multi-annual cycles to be reduced, and deterministic models to cease to exhibit multi-annual dynamics) are very robust to changes in parameters. Results are qualitatively similar for a broad range of biting and transmission rates, when the seasonal forcing is increased to 10% and reduced to 1%, when the seasonality is modeled as changes in mosquito birthrates, for a 2-vector/1-host system, and with an alternative formulation of the form of frequency dependence. Additionally, due to the intrinsic coupling between mosquito and primate, the observed dynamics in the mosquitoes are nearly identical to those in the primates.

## Discussion

Isolation of sylvatic dengue strains from humans in Senegal demonstrates cross-species dengue transmission from non-human primates to humans [1], [5], [9], [10], and viral isolations from mosquitoes, humans and non-human primates suggest a synchronous multi-annual cycle. It is unclear how important each primate host is in supporting sustained transmission and how the transmission cycles in each host affect transmission dynamics in others. We used deterministic and stochastic models of multiple primate hosts and multiple vectors to explore two questions about the observed dynamics in Senegal: 1) in the presence of multiple primate hosts with variation in population turnover, why is a single period observed in dengue incidence dynamics rather than multiple periods? and 2) can cycling in susceptibility among the multiple primate hosts from which dengue has been isolated explain the eight-year period the observed incidence of dengue? We found that even small amounts of coupling between species by cross-species biting of vectors can synchronize incidence in separate primate populations and that eight-year oscillations may be driven by cycles of immunity. Inclusion of a constant rate of infectious introduction lowers the length of the periods. Stochastic formulations of the model including infectious introduction exhibit eight-year oscillations, however, these models that show these dynamics exhibits reduced synchrony compared to empirical observations. Stochastic introductions of infectious individuals likely provide the “spark” of infection to ignite an epidemic once a sufficient number of susceptible primates have accumulated.

This is the first effort that we know of to characterize the dynamics of transmission models of dengue that incorporate multiple hosts and vectors. We found that in our coupled systems the species with the highest birth rate drove the epidemic dynamics in regions where its value of 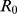 was greater than one. Time series showing long-period multi-annual dynamics were observed in large regions of realistic parameter space, where the high-birthrate (small) primate out numbered the low-birthrate (large) primate. In this region, the low-birthrate primate drove the isolation period with spillover into the high-birthrate primate (Figure 4). We found that long period multi-annual dynamics were observed only in regions where incidence during troughs reached small levels (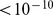). Therefore, the model suggests that dengue may undergo local extinctions in southeastern Senegal and depend upon periodic reintroductions from other parts of the country and west Africa. However, the effective population sizes of each primate and mosquito species are unknown, and therefore qualitative inferences may be made from our deterministic model. It may also be that an unobserved reservoir species exists that has drastically different temporal dynamics in its population and/or population turnover.

It is not entirely surprising that when we include a constant introduction of infectious individuals, multi-annual periodicities disappear. Including a constant rate of introduction is analogous to coupling the current primate-mosquito systems with a primate species with low amplitude, annual dengue outbreaks. Similar disruption of long period cycles has been demonstrated in deterministic models when populations with internal transmission are coupled to external infectious populations via mass action or immigration [44], [45]. Our models that did exhibit multi-annual dynamics with constant introduction show an increased role of stochasticity and reduced synchrony. Additional studies must be performed to characterize mechanisms that may dominant stochastic elements of our system including weather effects, migration and spatial interaction between primate populations and population sizes and structuring of both primate and vector species. Occasional reintroductions from surrounding populations that occur at long intervals might help explain the persistence of disease over long time scales and the long period dynamics.

The observed dynamics may reflect a bias in the surveillance techniques used to gather data that happen to undersample smaller outbreaks of dengue or be due to secular changes in collection methods over time. However, these hypotheses are not completely supported by the relative abundance of yellow fever and chikungunya isolates collected over the same time period and harvested from the same mosquitoes, and there is no evidence of significant secular changes in collection methods [2], [46]. This potential for bias is a fundamental limitation of this work: there is a lack of available data to accurately estimate parameters and build models. We have a narrow empirical window through which to view the complex system at work. More detailed serosurveys need to be conducted in both humans and other non-human primates as well as a quantitative measure of the off-diagonal biting rates of the various mosquito species and the population densities of the non-human primates in Senegal. The deterministic SIR model presented here, whose purpose was to give qualitative predictions as to the actual behavior of the system, assumes a well-mixed population. Importantly, our deterministic model does not allow for stochastic extinction or non-constant importation. A stochastic formulation of the model shows long-period cycles, but with reduced synchrony, and deserves a more detailed treatment.

This study makes qualitative predictions and generates empirically-testable hypotheses about the fundamental role of large and small primates in the sylvatic cycle of dengue virus in Senegal. It makes predictions, robust to large perturbations in parameters, that the coupling of primate-mosquito systems causes synchrony in outbreaks, and demonstrates that long period dynamics may be explained by cycling in immunity with stochastic introductions. An accurate and thorough understanding of the sylvatic cycle of dengue, including the roles of the various primate species in transmission, may allow prediction of epidemics and lessen the impact on humans living in rural and urban areas. Knowledge of the sylvatic cycle is especially important given evidence of recent introductions of sylvatic dengue into human populations and the potential these primate species have as reservoirs for dengue in post-vaccination scenarios. The current study is a step forward in the understanding of the determinants of sylvatic dengue transmission dynamics.

## Supporting Information

Supporting Information S1
**Supplemental information.** Supplemental Information includes: Model equations, analytical derivation of 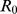, and many additional parameter explorations.(PDF)
