# Supplementary material for: Synchrony of Sylvatic Dengue Isolations: A Multi-Host, Multi-Vector SIR Model of Dengue Virus Transmission in Senegal
Source: PLoS Negl Trop Dis. 2012 Nov 29;6(11):e1928. doi: 10.1371/journal.pntd.0001928 (PMC3510077; doi:10.1371/journal.pntd.0001928)
Supplement: Supporting Information S1 — Supplemental information. Supplemental Information includes: Model equations, analytical derivation of , and many additional parameter explorations. (PDF) [file pntd.0001928.s001.pdf]

Supplementary Material for Synchrony of Sylvatic Dengue Isolations: A Multi-host, Multi-vector SIR Model of Dengue Virus Transmission in Senegal

Benjamin M. Althouse, Justin Lessler, Amadou A. Sall, Mawlouth Diallo, Kathryn A. Hanley, Douglas M. Watts, Scott C. Weaver, Derek A. T. Cummings

Model Equations

Our deterministic SIR model extends a framework presented in Keeling and Rohani (2008). The model equations are as follows, where S, I, and R denote the susceptible, infectious and recovered classes of each species and m_j indicates mosquito species j and p_i indicates primate species i (parameters are defined in Table 1 in the main text):

$$
\begin{aligned}
S'_{m_j}(t) &= \mu_{m_j}(S_{m_j}(t) + (1-\rho)I_{m_j}(t)) - \sum_i r_{m_j p_i}\beta_{p_i m_j}(t)I_{p_i}(t)S_{m_j}(t)/N_j(t) - \nu_{m_j}S_{m_j}(t) \quad (1)\\
I'_{m_j}(t) &= \mu_{m_j}\rho I_{m_j}(t) + \sum_i r_{m_j p_i}\beta_{p_i m_j}(t)I_{p_i}(t)S_{m_j}(t)/N_j(t) - \nu_{m_j}I_{m_j}(t) \quad (2)\\
S'_{p_i}(t) &= \mu_{p_i}N_{p_i}(t) - \sum_j r_{m_j p_i}\beta_{m_j p_i}(t)I_{m_j}(t)S_{p_i}(t)/N_j(t) - \nu_{p_i}S_{p_i}(t) \quad (3)\\
I'_{p_i}(t) &= \iota N_{p_i}(t) + \sum_j r_{m_j p_i}\beta_{m_j p_i}(t)I_{m_j}(t)S_{p_i}(t)/N_j(t) - \gamma_{p_i}I_{p_i}(t) - \nu_{p_i}I_{p_i}(t) \quad (4)\\
R'_{p_i}(t) &= \gamma_{p_i}I_{p_i}(t) - \nu_{p_i}R_{p_i}(t), \quad (5)
\end{aligned}
$$

with

$$
\begin{aligned}
\beta_{p_i m_j}(t) &= b_{p_i m_j}[1 + c_j \cdot \cos(t * 2\pi/365)] \quad (6)\\
\beta_{m_j p_i}(t) &= b_{m_j p_i}[1 + c_j \cdot \cos(t * 2\pi/365)] \quad (7)\\
N_{m_j} &= S_{m_j} + I_{m_j} \quad (8)\\
N_{p_i} &= S_{p_i} + I_{p_i} + R_{p_i} \quad (9)\\
N_j(t) &= \sum_k \left(\frac{r_{m_j p_k}}{\sum_k r_{m_j p_k}}\right) N_{p_k}. \quad (10)
\end{aligned}
$$

Figure S1 shows a diagram of the SIR model.

Analytical Calculation of R_0

To calculate R_0, we follow the formulation as laid out in Diekmann et al. [2]. We focus on the two-host, two-vector system, but it should be noted that this is easily extended to the n-host, n-vector system by adding the appropriate rows and columns to the proceeding matrices.

Following Diekmann, R_0 is the spectral radius of the Next Generation Matrix, $\mathbf{K}$, (i.e., $R_0 = \rho(\mathbf{K}) = \sup\{\mid \lambda \mid : \lambda \in \sigma(\mathbf{K})\}$ where $\sigma(\cdot)$ denotes the spectrum of matrix $\mathbf{K}$). We will decompose $\mathbf{K}$ into two matrices: $\mathbf{T}$, the *transmission matrix*, where $\mathbf{T}_{ij}$ is the rate at which infected individuals in state j infect individuals in state i; and Σ, the *transition matrix*, where Σ_{ij} is the rate an individual in state j transitions to state i. Diekmann et al. show that

$$\mathbf{K} = -\mathbf{E}^T\mathbf{T}\Sigma^{-1}\mathbf{E} \quad (11)$$

Where $\mathbf{T}^{-1}$ is the inverse of matrix $\mathbf{T}$ and $\mathbf{E}$ is a matrix of unit column vectors $\mathbf{e}_{ij}$ for all i such that the ith row of $\mathbf{T}$ is not identically zero.

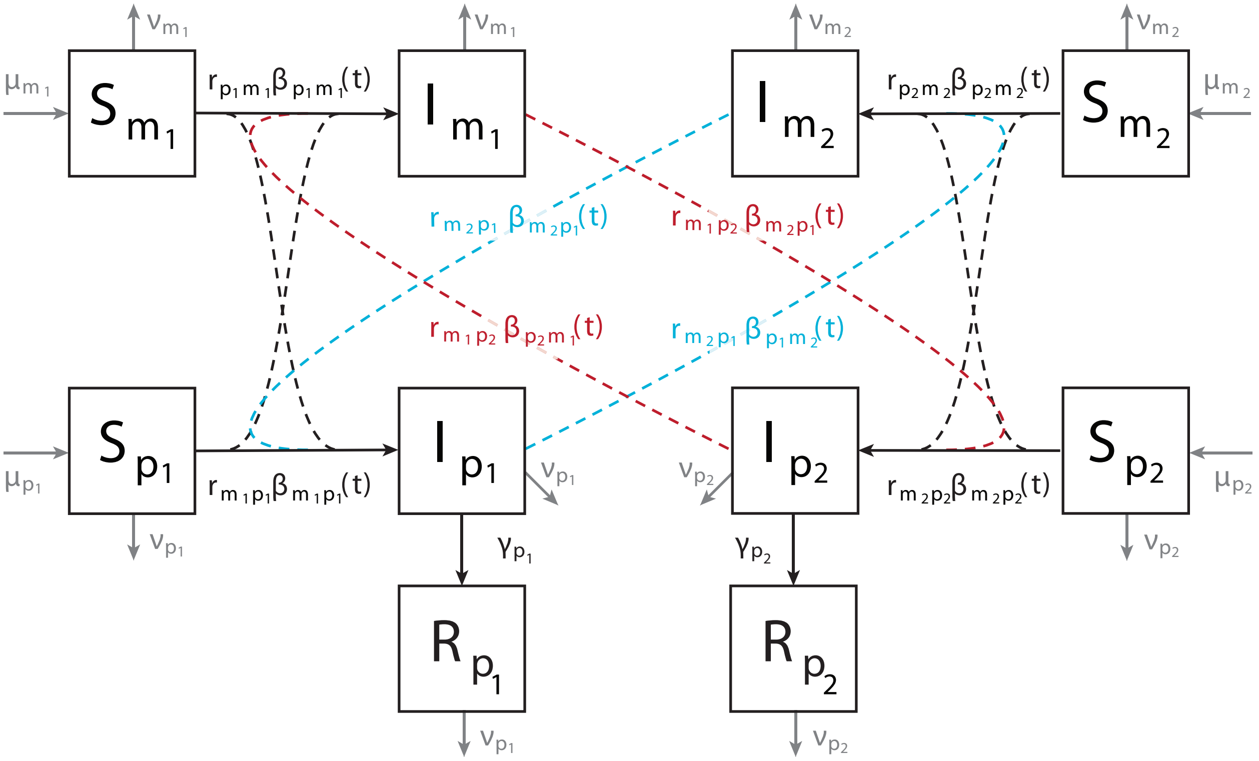

Figure S1: **Diagram of SIR Model**

We start by linearizing the system about the disease free equilibrium: $S^*_{m_j} = N_{m_j}$, $S^*_{p_i} = N_{p_i}$, with $I^*_{m_j} = I^*_{p_i} = R^*_{p_i} = 0$. Using the model equations given in the main text, we formulate the *infection subsystem* as:

$$I'_{m_j} = \sum_i r_{m_j p_i} b_{p_i m_j} I_{p_i} N_{m_j}/N_j - \nu_{m_j} I_{m_j} \tag{12}$$

$$I'_{p_i} = \sum_j r_{m_j p_i} b_{m_j p_i} I_m N_{p_i}/N_j - (\gamma_{p_i} + \mu_{p_i}) I_{p_i}, \tag{13}$$

where the (t)s have been dropped for clarity, the seasonal forcing functions ($\beta_{p_j m_i}(t)$) have been replaced by the baseline transmission probabilities ($b_{p_j m_i}$) and $N_j = \sum_i \left(\frac{r_{m_j p_i}}{\sum_i r_{m_j p_i}}\right) N_{p_i}$. We do not include vertical transmission.

For the infection subsystem above with two mosquito species, two primate species, the rate of transmission from primate i to mosquito j is given by

$$\frac{\partial}{\partial I_{p_i}}(I'_{m_j}) = r_{m_j p_i} \beta_{m_j p_i} \frac{N_{m_j}}{N_j}.$$

For $i = j = 1$, this is the (1,3) entry in $\mathbf{T}$. Continuing in this manner, we find the transmission matrix to be:

$$\mathbf{T} = \begin{pmatrix} 0 & 0 & r_{m_1 p_1} b_{m_1 p_1} \frac{N_{m_1}}{N_1} & r_{m_1 p_2} b_{m_1 p_2} \frac{N_{m_1}}{N_1} \\ 0 & 0 & r_{m_2 p_1} b_{m_2 p_1} \frac{N_{m_2}}{N_2} & r_{m_2 p_2} b_{m_2 p_2} \frac{N_{m_2}}{N_2} \\ r_{m_1 p_1} b_{p_1 m_1} \frac{N_{p_1}}{N_1} & r_{m_2 p_1} b_{p_1 m_2} \frac{N_{p_1}}{N_2} & 0 & 0 \\ r_{m_1 p_2} b_{p_2 m_1} \frac{N_{p_2}}{N_1} & r_{m_2 p_2} b_{p_2 m_2} \frac{N_{p_2}}{N_2} & 0 & 0 \end{pmatrix}. \tag{14}$$

As is expected, the diagonals of this matrix are 0: there is no direct transmission between species, all transmission is mediated by a vector. Next we calculate the transition matrix, Σ, where the (i, j) entry is the rate at which an individual in state j transitions to state i (excluding infection transitions). Since there are no transitions between infectious states in our infection subsystem, the transition matrix is a diagonal matrix with the entries equal to the demographic and recovery rates of change for each infected species:

$$\Sigma = \begin{pmatrix} -\nu_{m_1} & 0 & 0 & 0 \\ 0 & -\nu_{m_2} & 0 & 0 \\ 0 & 0 & -(\gamma_{p_1} + \mu_{p_1}) & 0 \\ 0 & 0 & 0 & -(\gamma_{p_2} + \mu_{p_2}) \end{pmatrix}. \tag{15}$$

This makes finding the inverse of Σ trivial, it is simply the reciprocal of each non-zero entry. Now, since none of the rows of $\mathbf{T}$ are identically zero, $\mathbf{E}$ is simply the 4-by-4 identity matrix and our next generation matrix (NGM), is $\mathbf{K} = -\mathbf{E}^T\mathbf{T}\Sigma^{-1}\mathbf{E} = -\mathbf{T}\Sigma^{-1} =$

$$\begin{pmatrix} 0 & 0 & \frac{r_{m_1p_1}b_{m_1p_1}N_{m_1}}{(\gamma_{p_1}+\mu_{p_1})N_1} & \frac{r_{m_1p_2}b_{m_1p_2}N_{m_1}}{(\gamma_{p_2}+\mu_{p_2})N_1} \\ 0 & 0 & \frac{r_{m_2p_1}b_{m_2p_1}N_{m_2}}{(\gamma_{p_1}+\mu_{p_1})N_2} & \frac{r_{m_2p_2}b_{m_2p_2}N_{m_2}}{(\gamma_{p_2}+\mu_{p_2})N_2} \\ \frac{r_{m_1p_1}b_{p_1m_1}N_{p_1}}{\nu_{m_1}N_1} & \frac{r_{m_2p_1}b_{p_1m_2}N_{p_1}}{\nu_{m_2}N_2} & 0 & 0 \\ \frac{r_{m_1p_2}b_{p_2m_1}N_{p_2}}{\nu_{m_1}N_1} & \frac{r_{m_2p_2}b_{p_2m_2}N_{p_2}}{\nu_{m_2}N_2} & 0 & 0 \end{pmatrix}. \tag{16}$$

Finding the eigenvalues of $\mathbf{K}$ can be done symbolically using a computer algebra program such as Mathematica. The final equation for R_0 is algebraically unwieldily, and is not presented here. Fortunately, it can easily be evaluated numerically and it helps characterize the behavior of the system under study and narrow the parameter space to key areas of interest.

Additional Parameter Exploration

The following sections report the results of exploring additional parameters not explored in the main text.

Inclusion of Constant Introduction of Infection

The addition of a constant rate of introduction of infected primates into both populations causes the multi-annual cycles to shorten to periods of a maximum of 4 years. Supplemental Figure S2 is identical to Figure 4 in the main text, but with a constant introduction rate of $1/100,000 \cdot N$ per year.

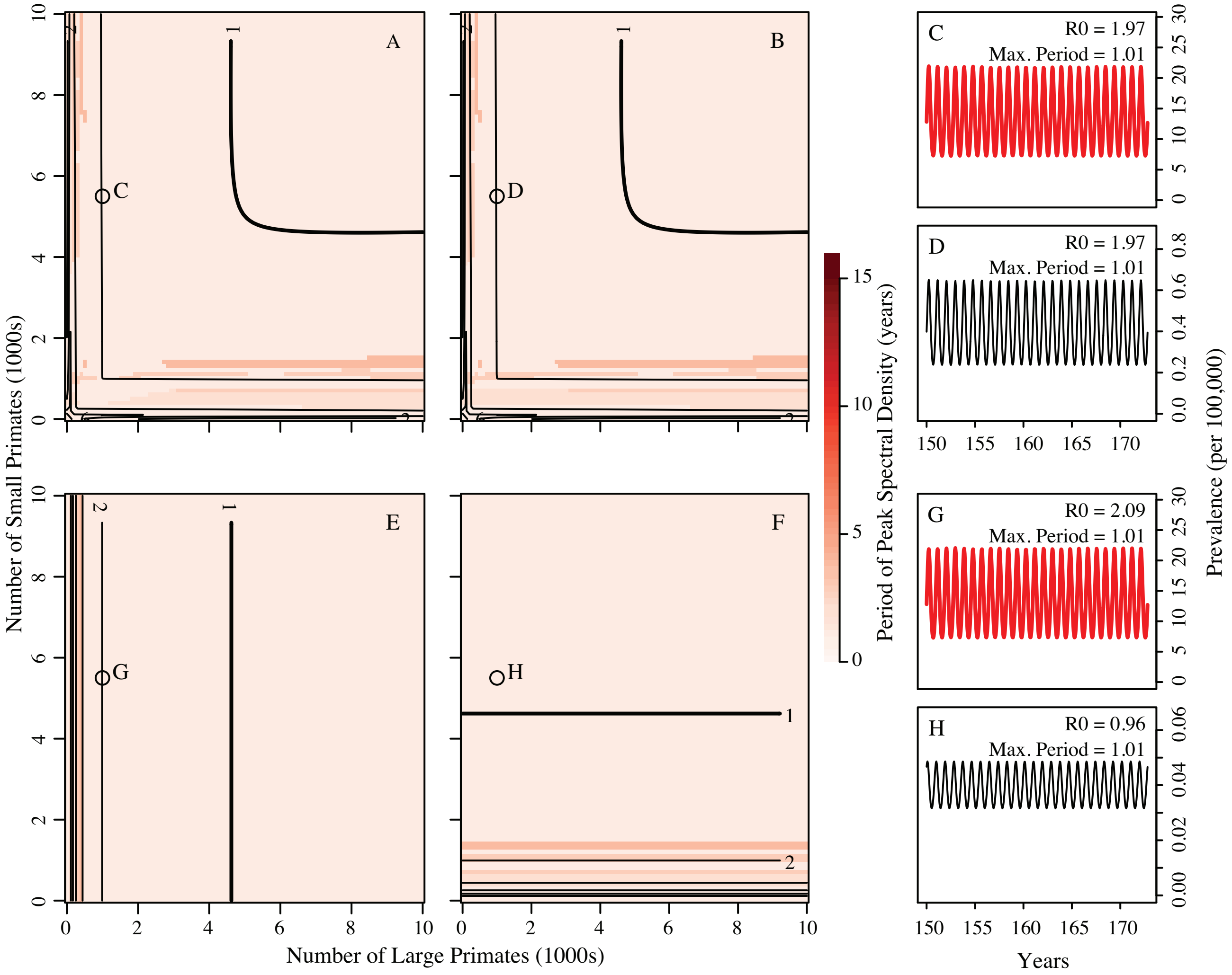

Figure S2: **Prevalence in Large and Small Primates in the Coupled and Uncoupled Systems with Constant Introduction** Figure shows the effect of adding a constant introduction of infection to the systems. Panels A and B are for the large and small primates, respectively, coupled at 1/500th of baseline, E and F are large and small primates uncoupled. Panels C, D, G and H are time series for both large and small primates in the coupled and uncoupled systems ($N_{p_1} = 1000$ and $N_{p_2} = 5500$). Parameters are: $1/\mu_{p_1} = 60$, $1/\mu_{p_2} = 15$, $1/\gamma_{p_1} = 1/\gamma_{p_2} = 4$, $b_{p_1 m_1} = b_{m_1 p_1} = b_{p_2 m_2} = b_{m_2 p_2} = 0.15$, $c_j = 0.05$, and $N_{m_j} = 25000$, $1/100,000 \cdot N$ per year rate of infection introduction.

Synchrony

Supplemental Figures S3 and S4 show coupling holding over a broad range of parameter values. Figure S3 shows the correlation between large and small primate prevalence time series and demonstrates that coupling causes phase synchrony over a broad range of parameters, and Figure S4 shows the effects of coupling hold under very small cross-species biting rates.

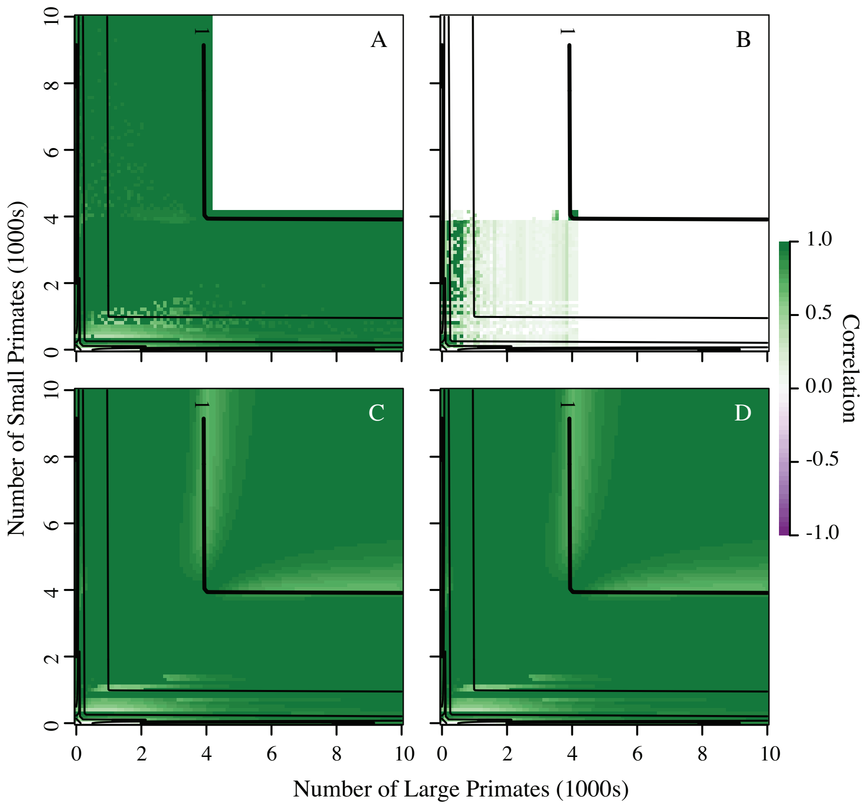

Figure S3: **Synchrony of prevalence in Coupled Systems Over a Range of Parameter Values with and without Constant Introduction** This figure shows the correlation between the two primate species in the coupled (A & C) and uncoupled (B & D) systems, with other parameters held fixed. Panel A is coupled at 1/500th of baseline without constant introduction of $1/100000 \cdot N$, B is uncoupled without introduction, C is coupled at 1/500th with constant introduction and D is uncoupled with introduction. The other parameter values are: $1/\mu_{p_1} = 60$, $1/\mu_{p_2} = 15$, $1/\gamma_{p_1} = 1/\gamma_{p_2} = 4$, $b_{p_1 m_1} = b_{m_1 p_1} = b_{p_2 m_2} = b_{m_2 p_2} = 0.15$, $c_j = 0.05$, and $N_{m_j} = 25000$, and $N_{p_1} = N_{p_2} = 1000$.

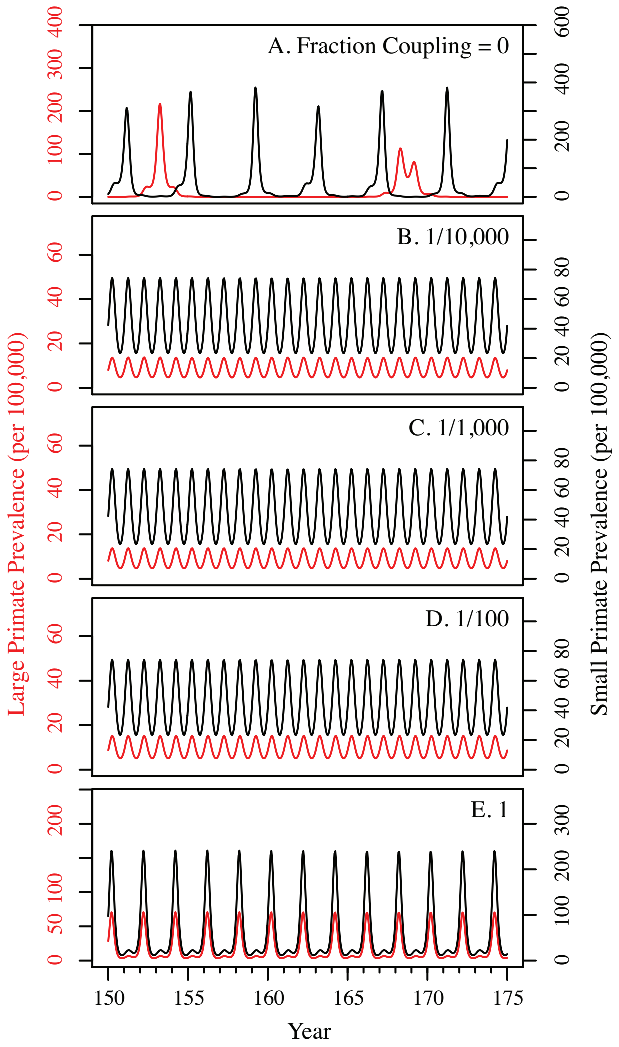

Figure S4: **Example Time Series with Increasing Coupling without Constant Introduction** This figure shows the effect of increasing off-diagonal biting rates ($r_{m_2p_1} = r_{m_1p_2}$), with other parameters held fixed. Panel A is uncoupled, panels B, C, D and E are coupled at 1/10,000th, 1/1,000th, 1/100th and 1 of the on-diagonal biting rates ($r_{m_1p_1} = r_{m_2p_2}$), respectively. Other parameter values are: $1/\mu_{p_1} = 60$, $1/\mu_{p_2} = 15$, $1/\gamma_{p_1} = 1/\gamma_{p_2} = 4$, $b_{p_1m_1} = b_{m_1p_1} = b_{p_2m_2} = b_{m_2p_2} = 0.15$, $c_j = 0.05$, and $N_{m_j} = 25,000$, and $N_{p_1} = 2,100$, $N_{p_2} = 1,500$.

Mosquito Dynamics

Supplemental Figures S5 and S6 are analogous to Figures 4 and 5 in the main text and show the transmission dynamics in the mosquito populations and highlight regions in parameter space where long period cycles occur. We find long period cycles in the coupled system when R_0 for the primary mosquito for the larger primate ("large primate mosquito") is less than one. Addition of a constant rate of introduction removes the long period cycles (results not shown).

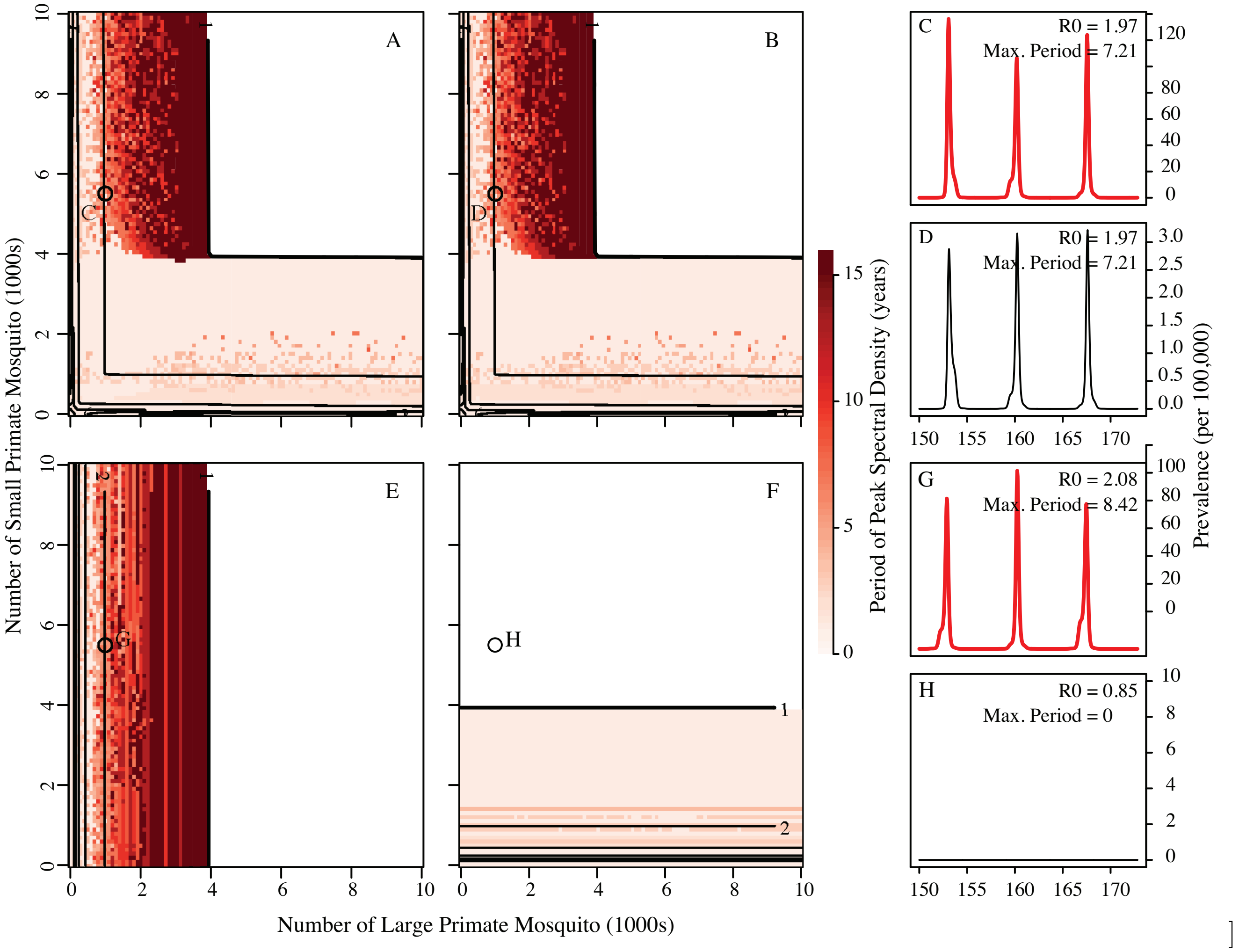

Figure S5: **Prevalence in Primary Mosquitoes for Large and Small Primates in the Coupled and Uncoupled Systems without Constant Introduction** Panels A and B show results for models with coupling, E and F for uncoupled models. Panel A and E characterize the dynamics of dengue in the large primate mosquito species, B and F, dengue dynamics in the small primate mosquito species. Coupled models (A, B, C and D) are coupled at 1/500th of the on-diagonal biting rates. Panels C, D, G and H show time series for large (C, G) and small primate mosquitoes (D, H) with parameters indicated by the circles in panels A, B, E and F ($N_{p_1} = 1,000$ and $N_{p_2} = 5,500$). The only parameter difference between panels A–D and panels E–H are the off-diagonal biting rates. Other parameters are: $1/\mu_{p_1} = 60$, $1/\mu_{p_2} = 15$, $1/\gamma_{p_1} = 1/\gamma_{p_2} = 4$, $b_{p_1 m_1} = b_{m_1 p_1} = b_{p_2 m_2} = b_{m_2 p_2} = 0.15$, $c_j = 0.05$, and $N_{m_j} = 25,000$, $j = \{1,\ 2\}$.

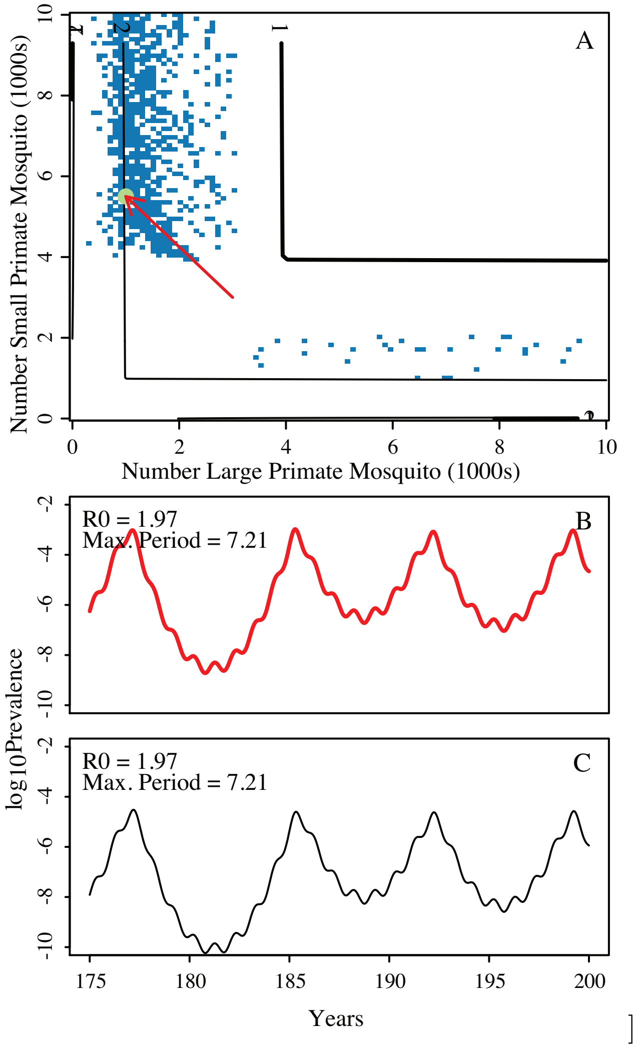

Figure S6: **Example Time Series of Long-Period Isolations in Mosquitoes** This figure indicates the regions of model parameter space that exhibit multiannual dynamics consistent with the observed periodicity of mosquito isolations of dengue in Senegal. The blue dots highlight areas of panel A in Figure 4 in the main text where the Fourier spectrum has a maximum between 5 and 12 years. The figure also shows an example time series of long-period, synchronized cycles in the large primate mosquito (panel B) and small primate mosquito (panel C). The arrow and green dot indicate the position in parameter space that was used to generate the time series in panels B and C. Here, $N_{p_1} = 1,000$ and $N_{p_2} = 5,500$ are coupled at 1/500th of the on-diagonal biting rates. Other parameter values are: $1/\mu_{p_1} = 60$, $1/\mu_{p_2} = 15$, $1/\gamma_{p_1} = 1/\gamma_{p_2} = 4$, $b_{p_1m_1} = b_{m_1p_1} = b_{p_2m_2} = b_{m_2p_2} = 0.15$, $c_j = 0.05$, and $N_{m_j} = 25,000$, $j = \{1,\ 2\}$.

Biting Rates

Supplemental Figure S7 shows the effects of changing biting rates on the dynamics of the system. We find long period cycles in the coupled system when R_0 for the smaller primate is less than one. Addition of a constant rate of introduction removes the long period cycles (results not shown).

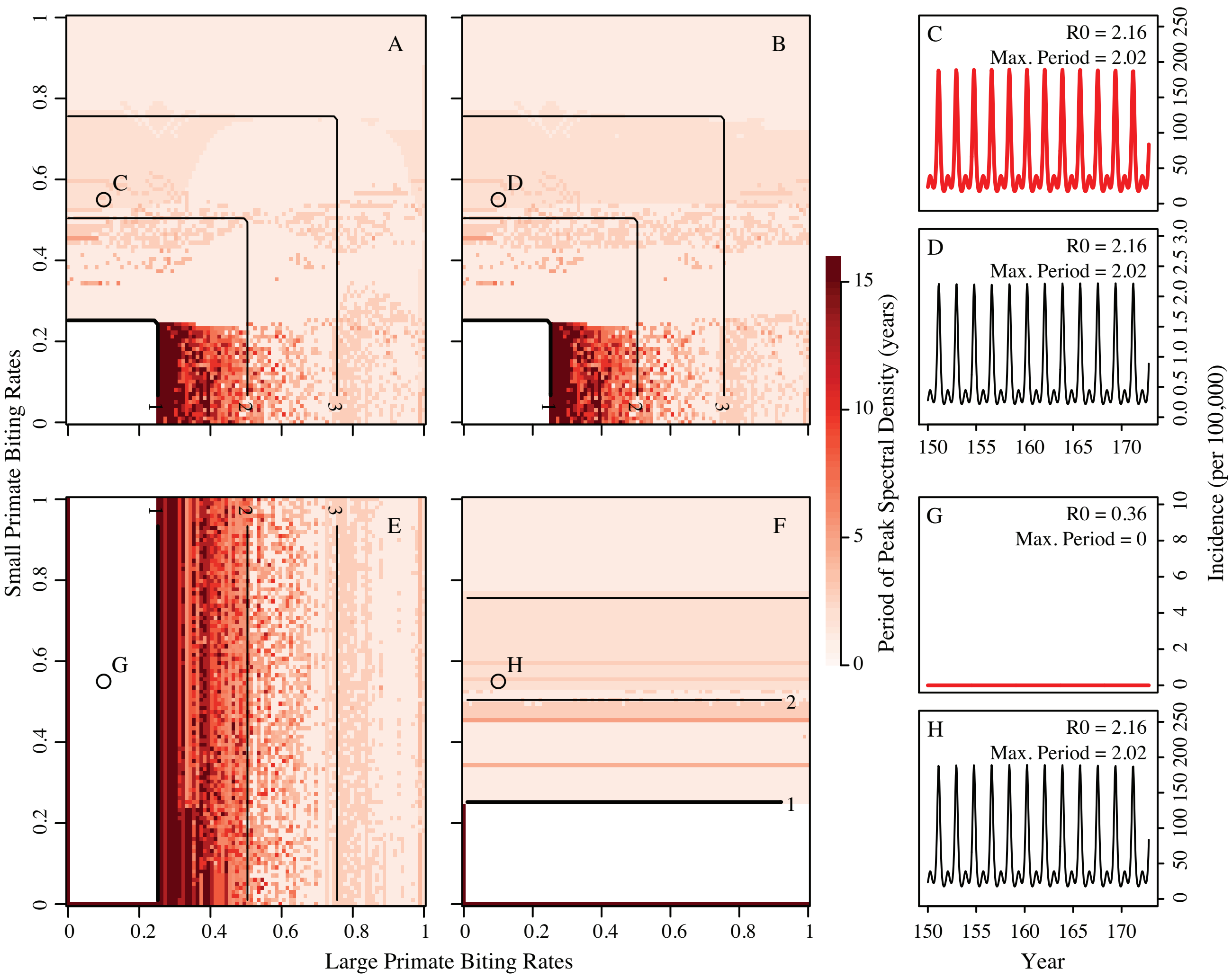

Figure S7: **Exploration of Mosquito Biting Rates on Large and Small Primates without Constant Introduction** This figure shows the effects of varying biting rates on the periodicities of the system. Panels A and B are for the large and small primates, respectively, coupled at 1/500th of baseline, E and F are large and small primates uncoupled. Panels C, D, G and H are example time series. The other parameter values are: $1/\mu_{p_1} = 60$, $1/\mu_{p_2} = 15$, $1/\gamma_{p_1} = 1/\gamma_{p_2} = 4$, $b_{p_1 m_1} = b_{m_1 p_1} = b_{p_2 m_2} = b_{m_2 p_2} = 0.15$, $c_j = 0.05$, and $N_{m_j} = 25000$, and $N_{p_1} = N_{p_2} = 1000$.

Transmission Rates

Supplemental Figure S8 shows the effects of changing transmission rates on the dynamics of the system. Again, we find long period cycles in the coupled system when R_0 for the smaller primate is less than one. Addition of a constant rate of introduction removes the long period cycles (results not shown).

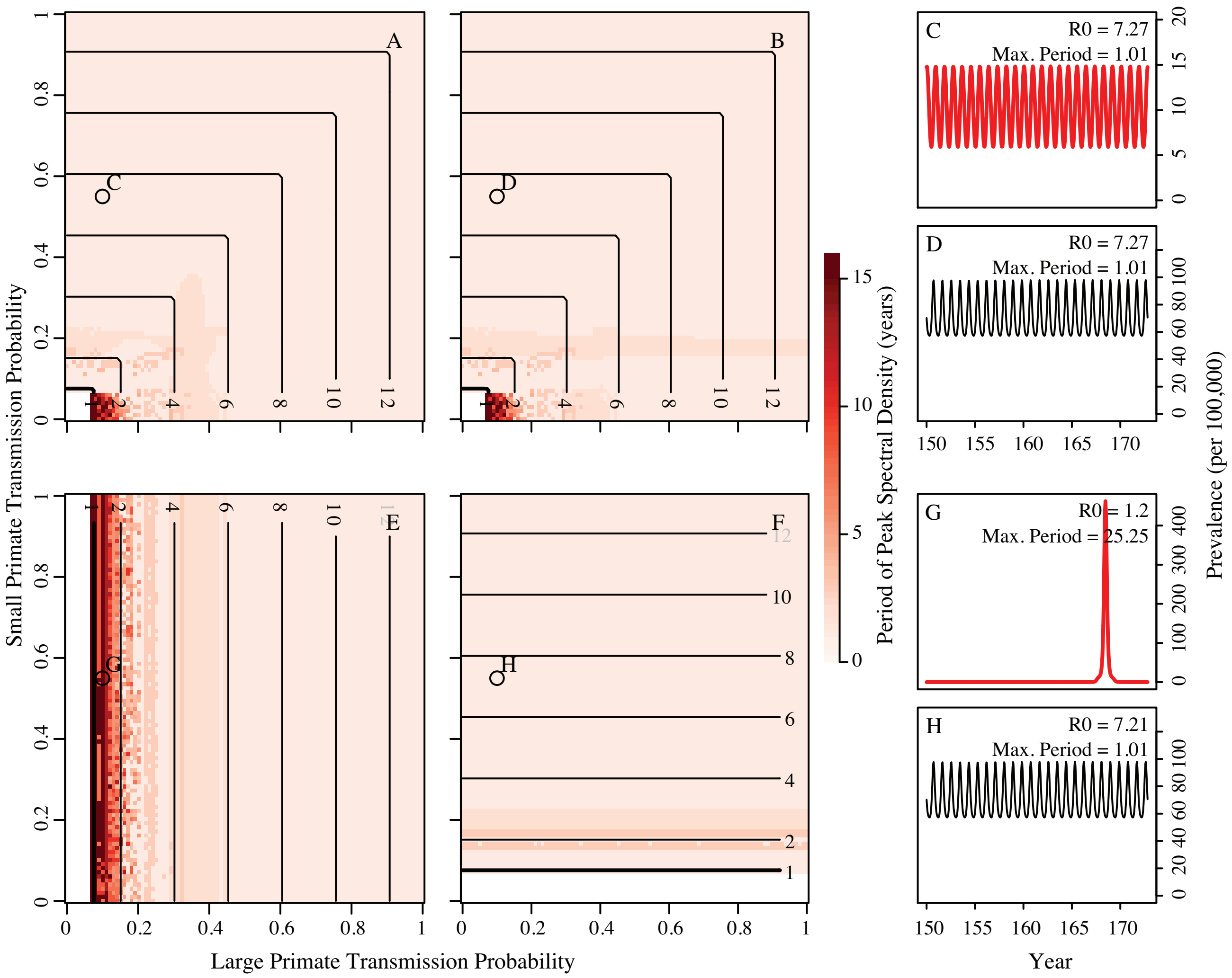

Figure S8: **Exploration of Mosquito Transmission Rates on Large and Small Primates without Constant Introduction** This figure shows the effects of varying transmission rates on the periodicities of the system. Panels A and B are for the large and small primates, respectively, coupled at 1/500th of baseline, E and F are large and small primates uncoupled. Panels C, D, G and H are example time series. The other parameter values are: $1/\mu_{p_1} = 60$, $1/\mu_{p_2} = 15$, $1/\gamma_{p_1} = 1/\gamma_{p_2} = 4$, $r_{p_i m_j} = 0.5$, $b_{p_1 m_1} = b_{m_1 p_1}$, $b_{p_2 m_2} = b_{m_2 p_2}$, $c_j = 0.05$, and $N_{m_j} = 25000$, and $N_{p_1} = N_{p_2} = 1000$.

Seasonal Forcing Rates

Supplemental Figure S9 shows the effects of increasing the seasonal forcing rates from 5% a year to 10% a year, and Supplemental Figure S10 shows the effects of chaining forcing to 1% a year. The results are qualitatively similar to those found in the main text. Addition of a constant rate of introduction removes the long period cycles (results not shown).

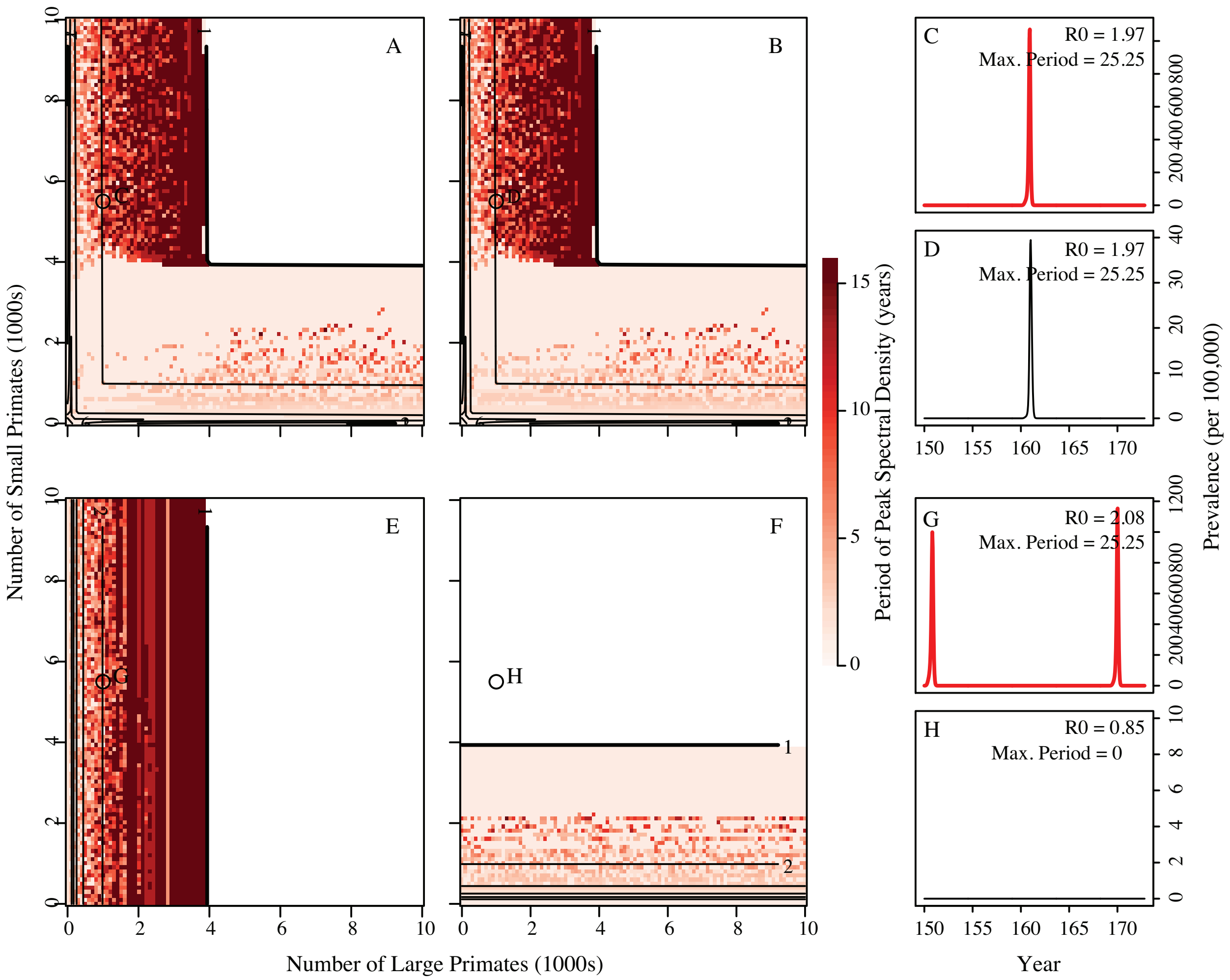

Figure S9: **Effects of High Seasonal Forcing** Top panels (A, B, C and D) are coupled at 1/500th of the on-diagonal biting rates. Bottom panels (E, F, G and H) are uncoupled. Panels C, D, G and H are time series for both large and small primates in the coupled and uncoupled systems. Seasonal forcing, $c_j = 0.1$. Other parameters are: $1/\mu_{p_1} = 60$, $1/\mu_{p_2} = 15$, $1/\gamma_{p_1} = 1/\gamma_{p_2} = 4$, $b_{p_1 m_1} = b_{m_1 p_1} = b_{p_2 m_2} = b_{m_2 p_2} = 0.15$, and $N_{m_j} = 25000$.

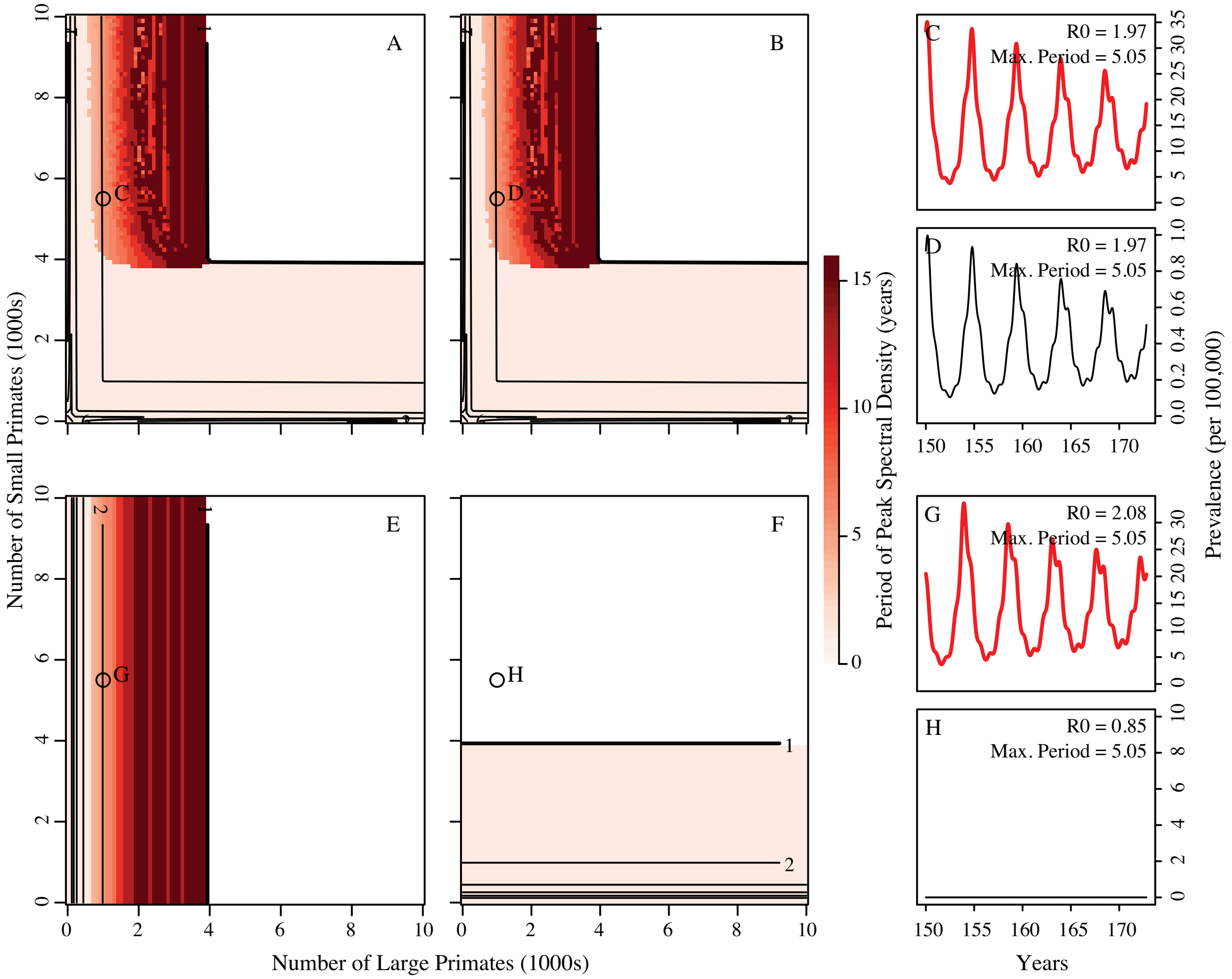

Figure S10: **Effects of Low Seasonal Forcing** Top panels (A, B, C and D) are coupled at 1/500th of the on-diagonal biting rates. Bottom panels (E, F, G and H) are uncoupled. Panels C, D, G and H are time series for both large and small primates in the coupled and uncoupled systems. Seasonal forcing, $c_j = 0.01$. Other parameters are: $1/\mu_{p_1} = 60$, $1/\mu_{p_2} = 15$, $1/\gamma_{p_1} = 1/\gamma_{p_2} = 4$, $b_{p_1 m_1} = b_{m_1 p_1} = b_{p_2 m_2} = b_{m_2 p_2} = 0.15$, and $N_{m_j} = 25000$.

Alternative Formulation of Seasonal Forcing

Supplemental Figure S11 shows the effects of changing the seasonal forcing from the transmission probability, $\beta_{p_i m_j}(t) = b_{p_i m_j}[1 + c_j \cdot \cos(t * 2\pi/365)]$, to the mosquito birthrate,

$$\mu_{m_j}(t) \quad = \quad M_{m_j}[1 + c \cdot \cos(t * 2\pi/365)],$$

where M_{m_j} is the baseline birthrate for mosquito j ($= 1/7$ days), and c is the percent seasonality ($= 5\%$). The results are qualitatively similar to those found in the main text. Addition of a constant rate of introduction removes the long period cycles (results not shown).

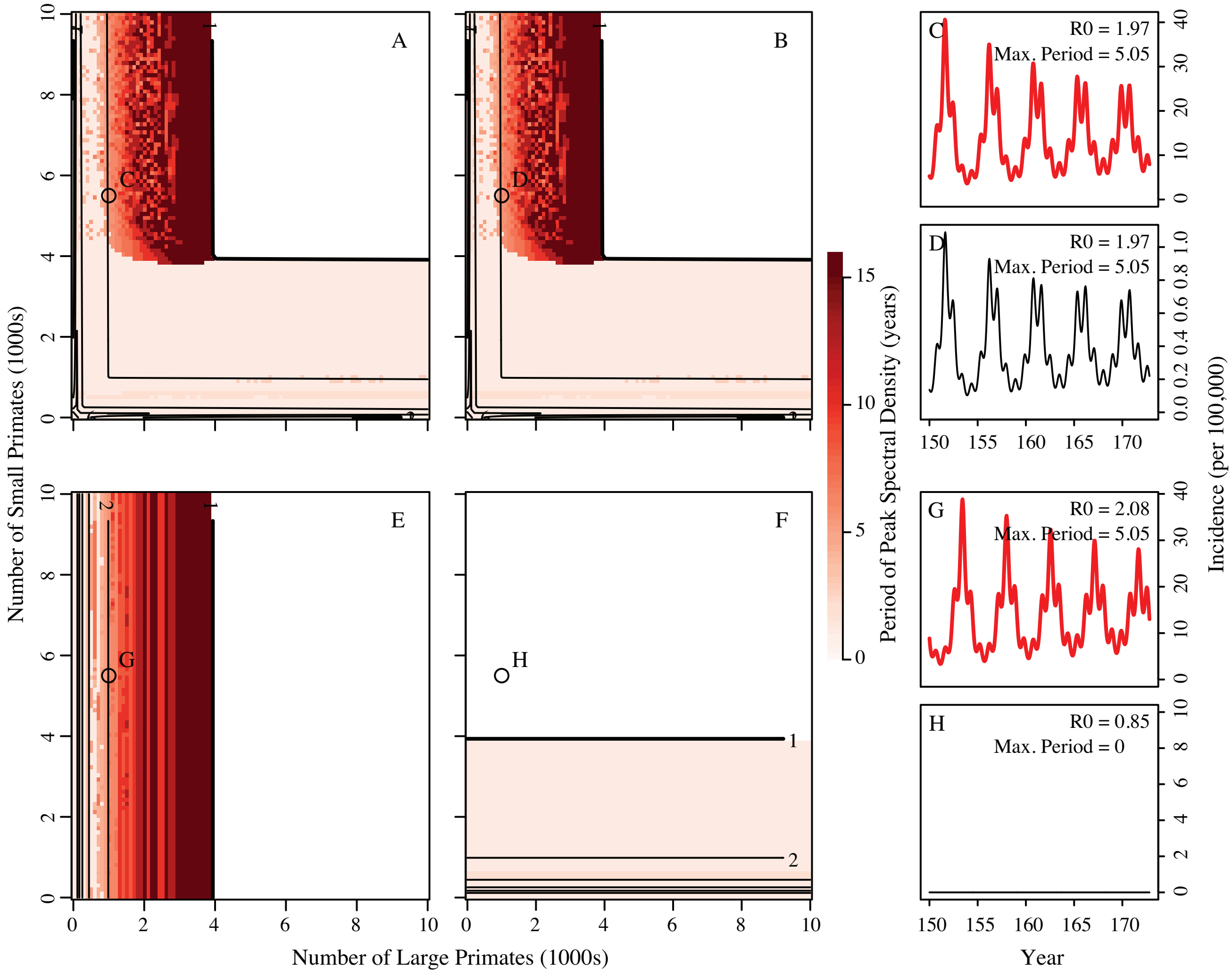

Figure S11: **Effects of a Seasonal Birthrate** Top panels (A, B, C and D) are coupled at 1/500th of the on-diagonal biting rates. Bottom panels (E, F, G and H) are uncoupled. Panels C, D, G and H are time series for both large and small primates in the coupled and uncoupled systems. Other parameters are: $1/\mu_{p_1} = 60$, $1/\mu_{p_2} = 15$, $1/\gamma_{p_1} = 1/\gamma_{p_2} = 4$, $b_{p_1 m_1} = b_{m_1 p_1} = b_{p_2 m_2} = b_{m_2 p_2} = 0.15$, $c_j = 0.05$, and $N_{m_j} = 25000$.

2-Vector/1-Host System

Supplemental Figure S12 shows the results of modeling a 2-vector, 1-host system. The results are qualitatively similar to those found in the main text. Addition of a constant rate of introduction removes the long period cycles.

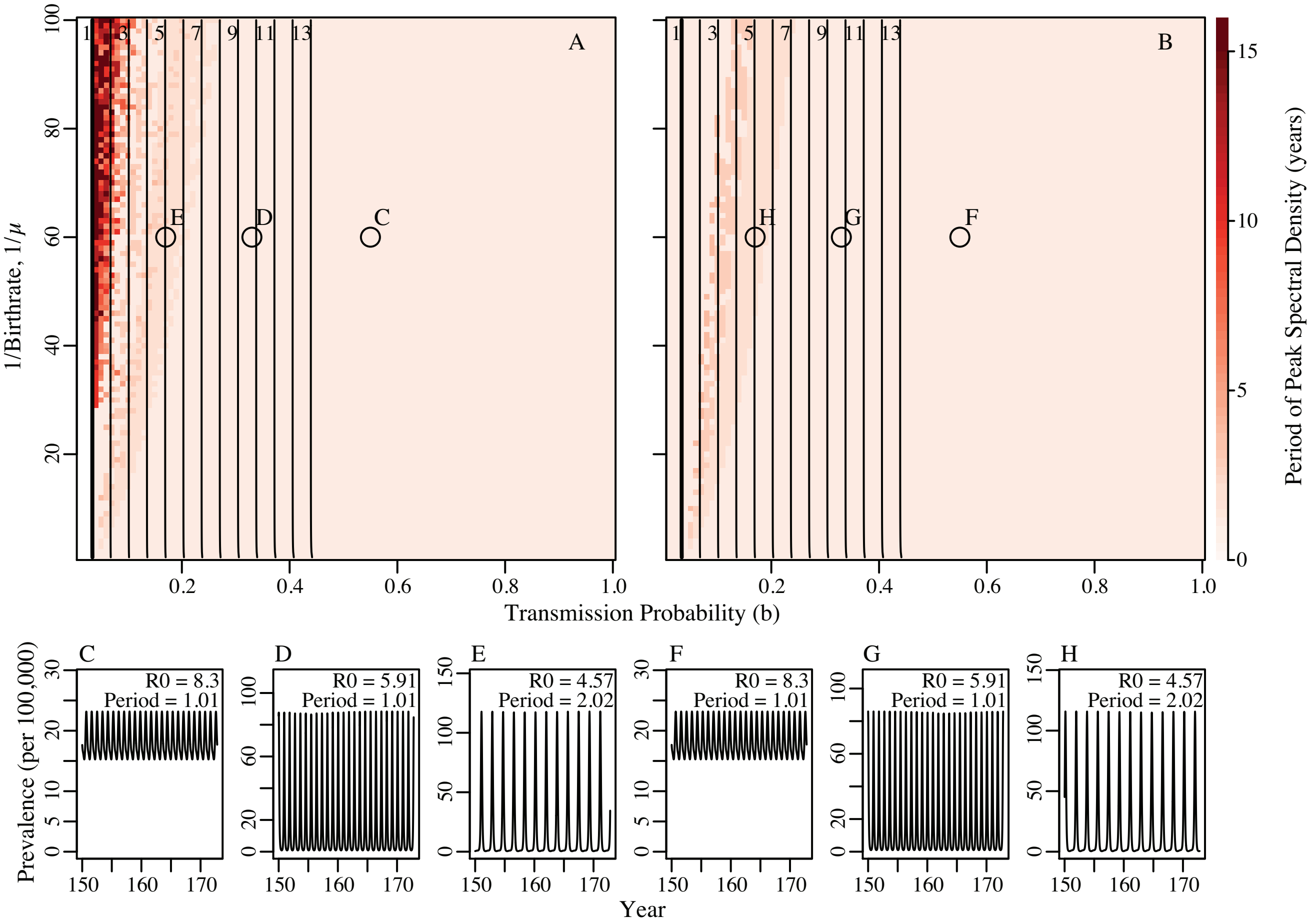

Figure S12: **2-Vector, 1-Host Systems with and without Constant Introduction** This figure displays the effects of changing transmission probabilities (x-axis) and 1/primate birth rates (y-axis). Panels A and B are heat maps of period of peak Fourier spectral densities for the host in the 2-host, 1-vector systems, with and without $1/100,000 \cdot N$ per year rate of infection introduction. Panels C-H are the corresponding example epidemic time series of prevalence (per 100,000). Circles indicate example time series below. Other parameters held fixed: $r_p = 0.5$, $1/\gamma_p = 4$, $c_j = 0.05$, $N_m = 25,000$, and $N_p = 1,000$.

Stochastic Model Without Coupling

Supplemental Figure S13 shows the results of a stochastic formulation of the model without coupling. In accordance with our hypotheses, stochastic realizations without coupling show little to no synchrony.

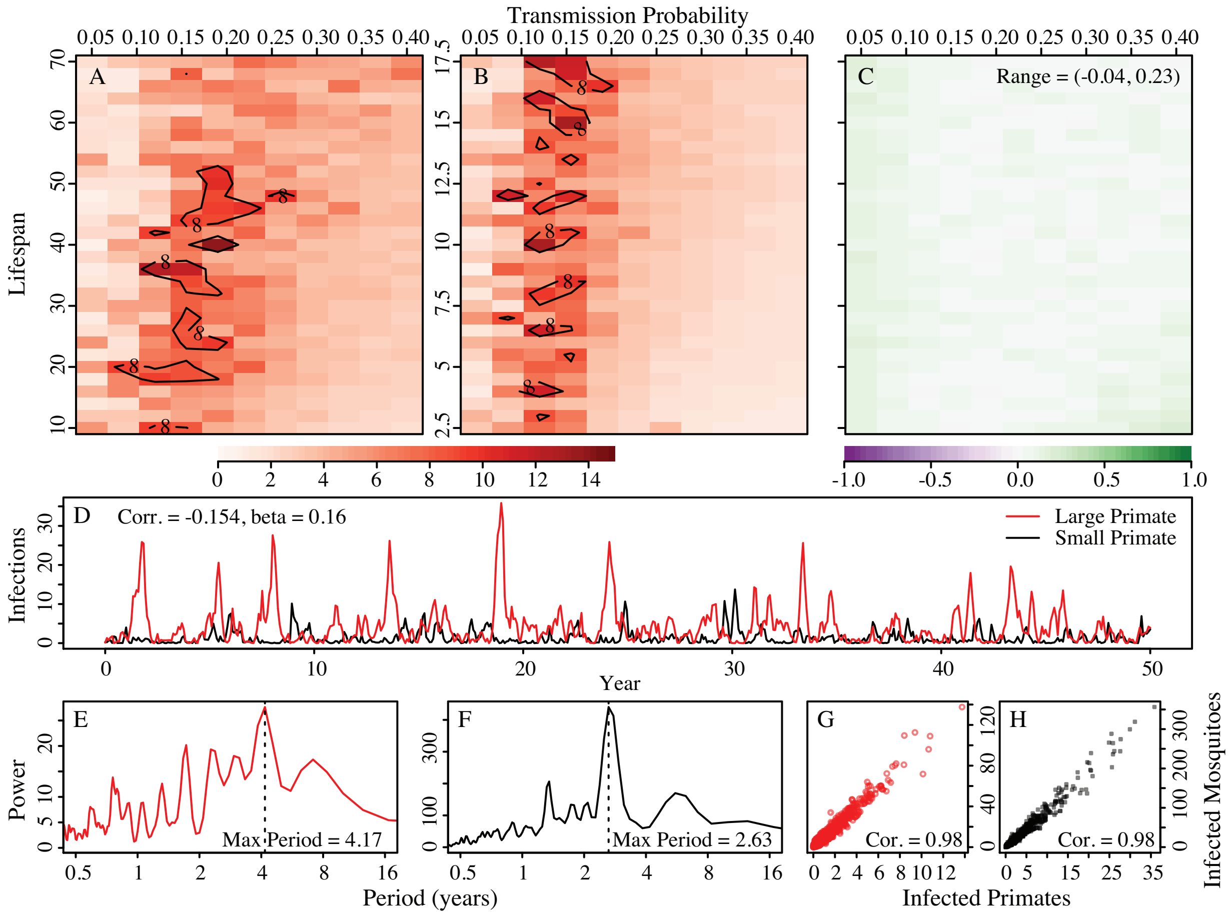

Figure S13: **Stochastic Formulation of the Model Without Coupling** Heatmap of the period of maximum Fourier spectra with corresponding example epidemic time series of prevalence. Panels A, B and C compare transmission probabilities (x-axis) and 1/birth rate (y-axis) for the large primate (A) and the small primate (B). Birthrates for the small primate are 1/4th of those of the large. Panel A shows periods of oscillations for large primates, B, periods of oscillations for small primates and C the correlation of the mean number of cases in a year (all panels are averaged over 25 runs). D is an example realization of the model with long periodicity. Fourier spectra for the large and small primate time series are shown in panels E and F, respectively. Panels G and H are scatterplots of the number of primate infections versus number of mosquito infections for the large and small primates and their corresponding mosquitoes, respectively. There is no coupling; other parameters are: $\mu_1 = 1/70$ and $\mu_2 = 1/17.5$, $\iota = 1/500$, $r_{p_i m_j} = 0.5$, $\beta_{p_i m_j} = 0.16$, $1/\gamma_{p_i} = 4$, $c_j = 0.05$, $N_{m_j} = 150,000$, and $N_{p_i} = 10,000$, $i = \{1,\ 2\}$, $j = \{1,\ 2\}$.

Alternative Formulation of Frequency Dependent Transmission

The force of infection for primate i as presented in the main text is $\sum_j r_{m_j p_i} \beta_{m_j p_i}(t) I_{m_j}(t)/N_j(t)$: the number of infectious bites from mosquito j to primate i, times the probability of mosquito j infecting primate i times the number of infectious mosquitoes j, divided by sum of all available primates weighted by the on– and off–diagonal biting rates. This models an extreme of mosquito behavior: the mosquito hones in on meals with great efficiency. Here we model the other extreme of poor efficiency where mosquitoes might have innate preferences but be confused by the environmental cues of other species (e.g. carbon dioxide, organic volatile body odors, air movement, heat) used to find preferred hosts, and feed on whatever host it first encounters [3]. This is modeled by replacing N_j with N, the total primate population size, in the force of infection.

There is little consensus on the formulation of this term in previous modeling studies. An examination of previous multi-host models (predominantly of West Nile Virus) reveals a divide between the "weighted" frequency dependence form (as presented in the main text) [4, 5, 6, 7, 8] and the "unweighted" frequency dependence form presented here [9, 10, 11, 12, 13, 14]. Additionally, several studies [15, 16, 17] have modeled the transmission in a density dependent manner for both hosts and vectors.

Supplemental Figures S14–S16 are analogous to those in the main text, but with the new force of infection. In the poor efficiency case we find qualitatively similar results. Again, we find long period cycles when transmission probabilities are low that are lost when constant introduction in included (Supplemental Figures S14 and S15). We also find the smaller primate dictating the periodicity of outbreaks when its $R_0 > 1$ (Supplemental Figure S16) and again, the long period cycles are removed when constant introduction is included (Supplemental Figure S17).

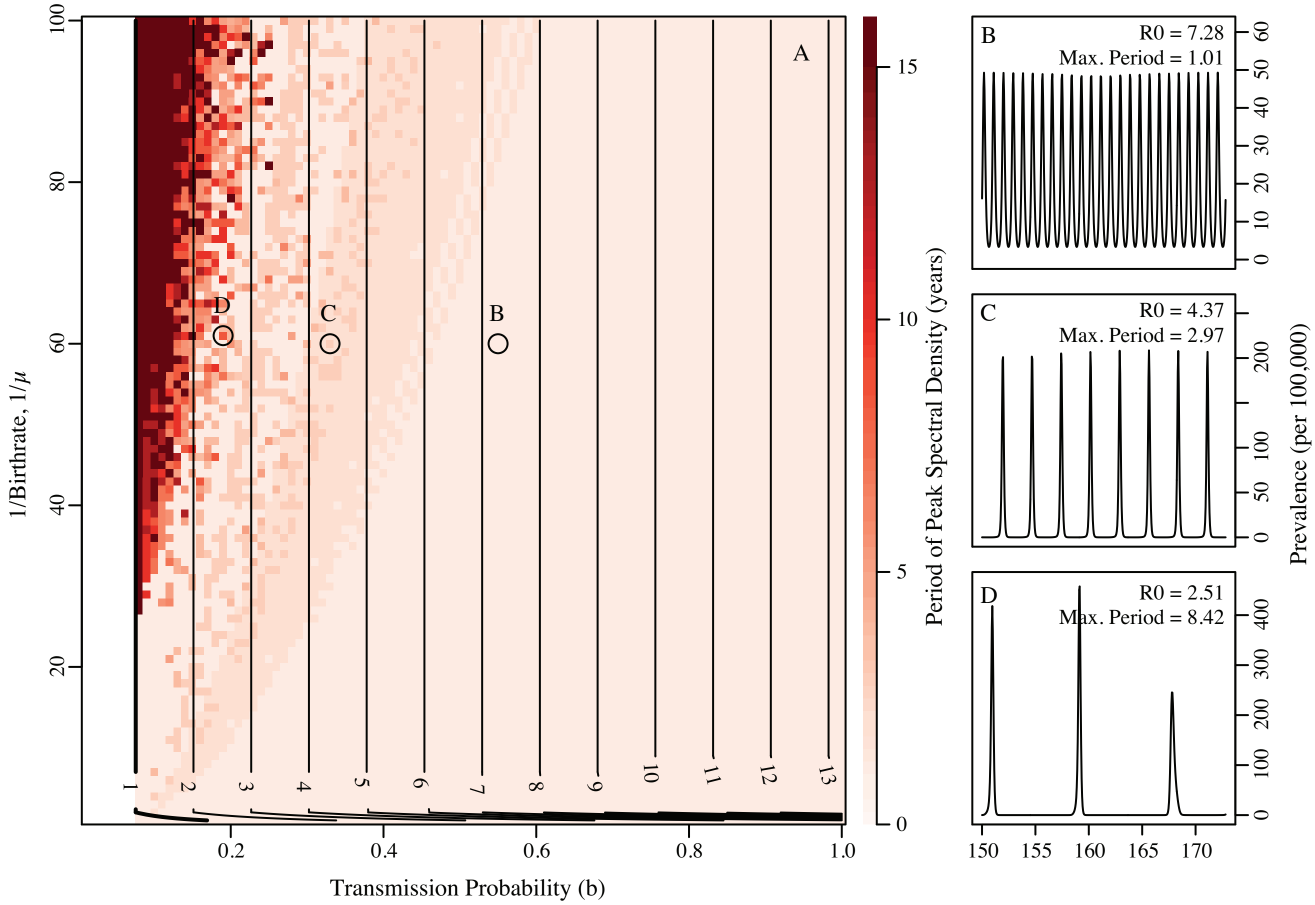

Figure S14: **Effect of Demographics on Model Dynamics without Constant Introduction and New Force of Infection** Heat map of period of maximum Fourier spectra peak (Panel A) with corresponding example epidemic time series of prevalence per 100,000 (Panels B, C and D). Figure compares transmission probabilities (x-axis) and 1/birth rate (y-axis). Circles indicate example time series on right. Other parameters held fixed: $r_p = 0.5$, $1/\gamma_p = 4$, $c_j = 0.05$, $N_m = 25000$, and $N_p = 1000$.

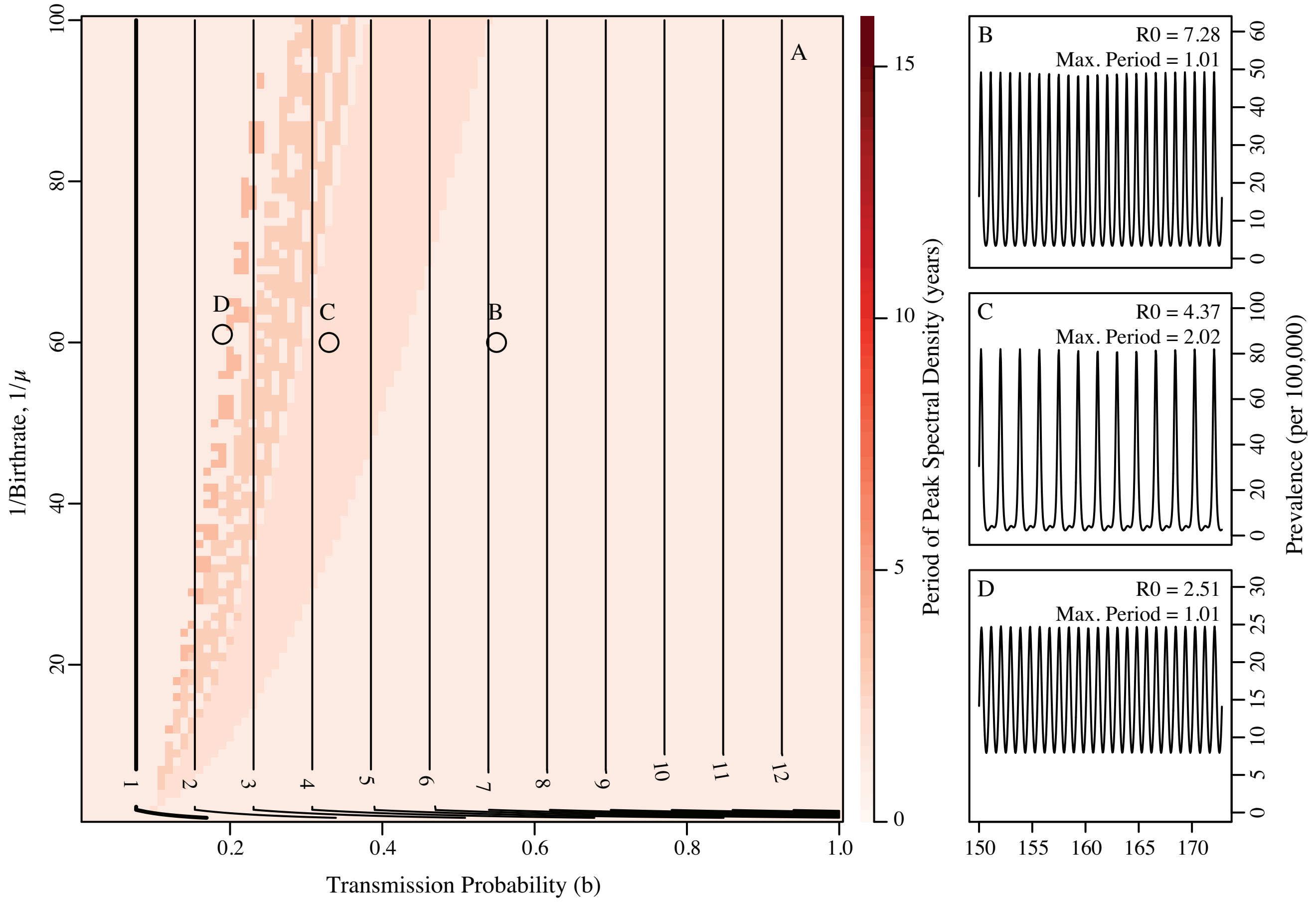

Figure S15: **Effect of Demographics on Model Dynamics with Constant Introduction and New Force of Infection** Heat map of period of maximum Fourier spectra peak (Panel A) with corresponding example epidemic time series of prevalence per 100,000 (Panels B, C and D). Same parameters as in Figure S14 with $1/100,000 \cdot N$ per year rate of infection introduction.

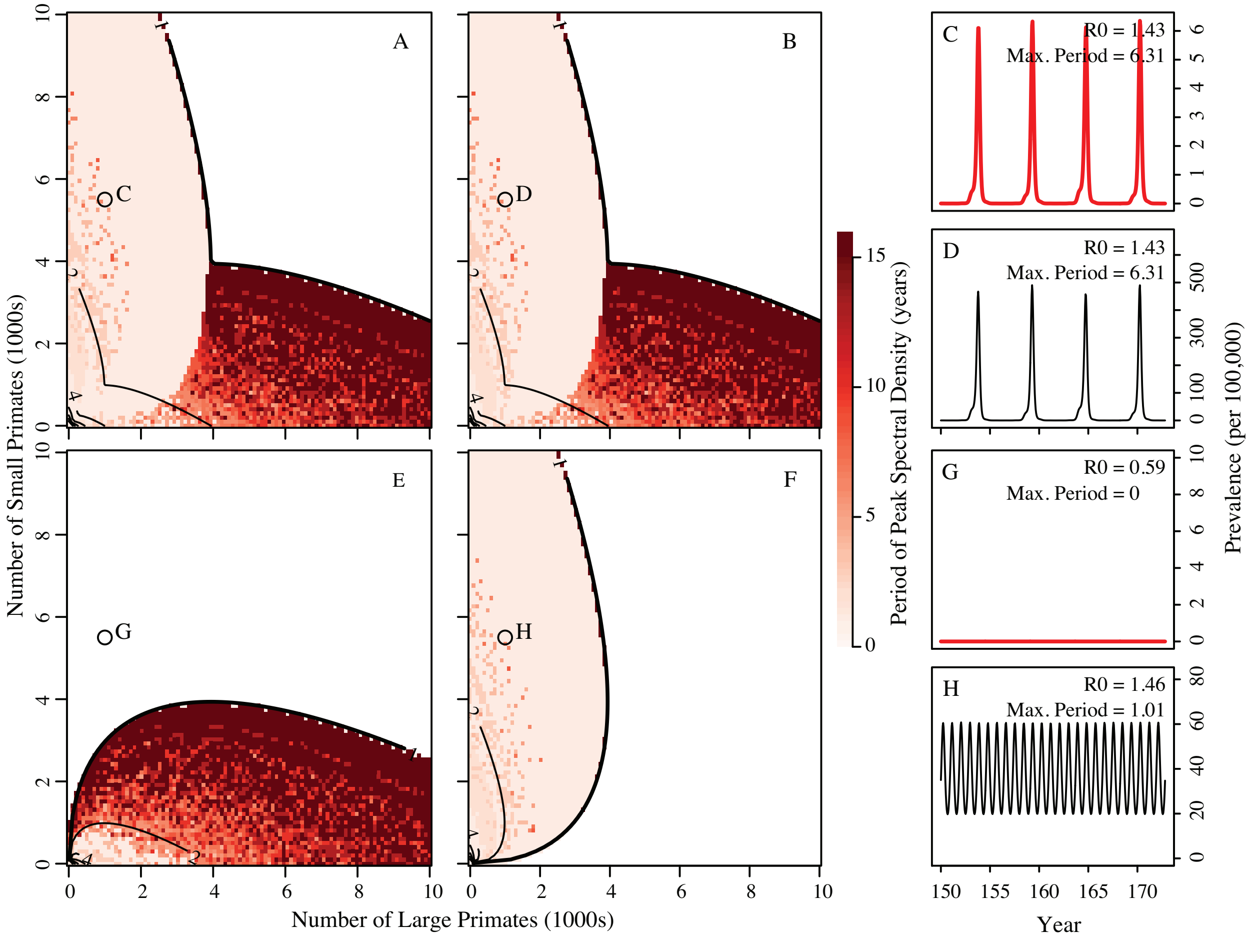

Figure S16: **Prevalence in Large and Small Primates in the Coupled and Uncoupled Systems without Constant Introduction and New Force of Infection** Top panels (A, B, C and D) are coupled at 1/500th of the on-diagonal biting rates. Bottom panels (E, F, G and H) are uncoupled. Note the dominance of the higher birth rate primate on the period of oscillations in the coupled simulations. This indicates that the higher birth rate species determines the period of epidemic oscillation only when its R_0 is greater than one (this area is delineated in panel F). Panels C, D, G and H are time series for both large and small primates in the coupled and uncoupled systems ($N_{p_1} = 1000$ and $N_{p_2} = 5500$). Other parameters are: $1/\mu_{p_1} = 60$, $1/\mu_{p_2} = 15$, $1/\gamma_{p_1} = 1/\gamma_{p_2} = 4$, $b_{p_1 m_1} = b_{m_1 p_1} = b_{p_2 m_2} = b_{m_2 p_2} = 0.3$, $c_j = 0.05$, and $N_{m_j} = 25000$.

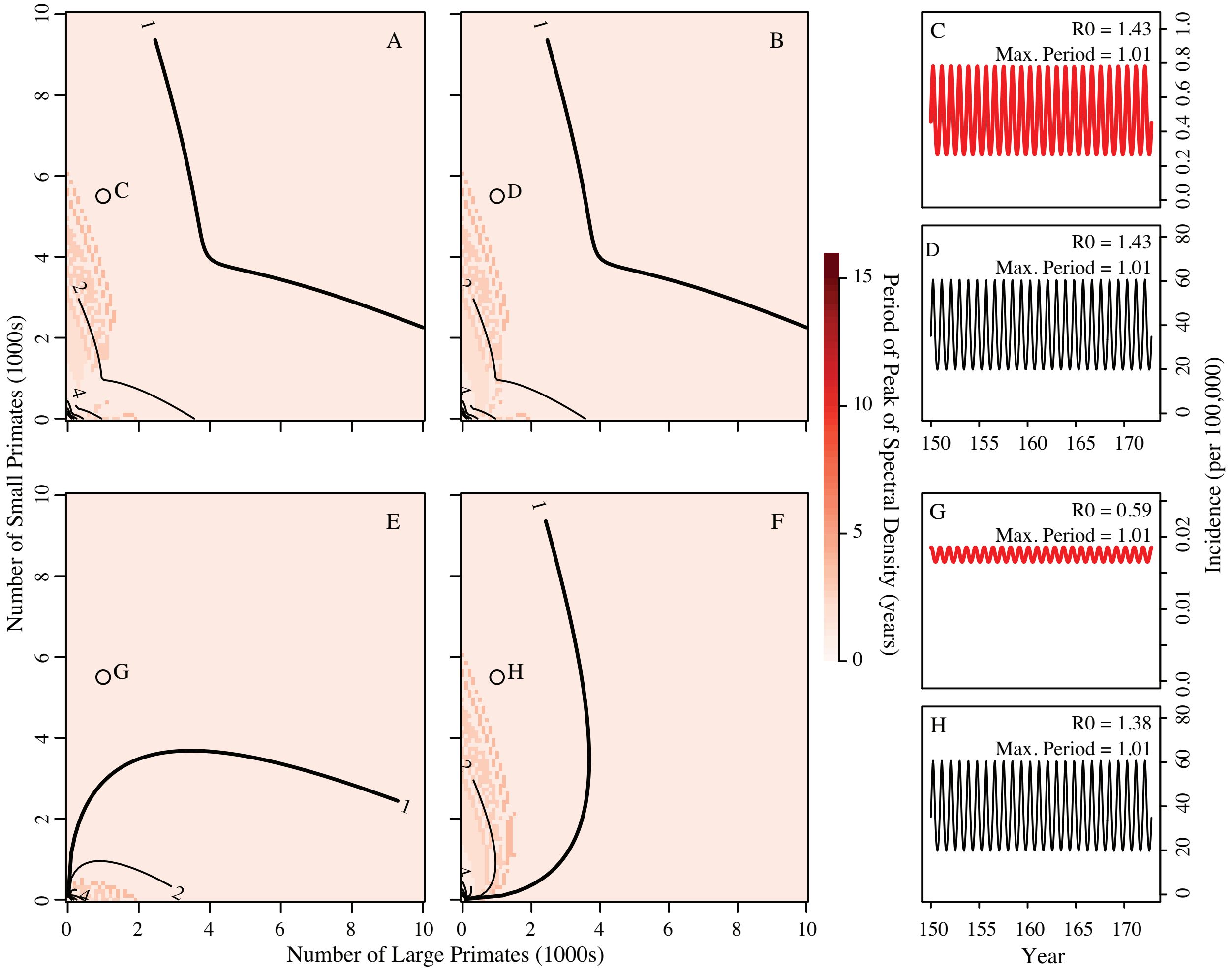

Figure S17: **Prevalence in Large and Small Primates in the Coupled and Uncoupled Systems without Constant Introduction** Top panels (A, B, C and D) are coupled at 1/500th of the on-diagonal biting rates. Bottom panels (E, F, G and H) are uncoupled. Panels 1 through 4 are time series for both large and small primates in the coupled and uncoupled systems ($N_{p_1} = 5500$ and $N_{p_2} = 1000$). Note the only parameter difference between panels A, B, 1 & 2 and panels C, D, 3 & 4 are the off-diagonal biting rates. Other parameters are: $1/\mu_{p_1} = 60$, $1/\mu_{p_2} = 15$, $1/\gamma_{p_1} = 1/\gamma_{p_2} = 4$, $b_{p_1m_1} = b_{m_1p_1} = b_{p_2m_2} = b_{m_2p_2} = 0.3$, $c_j = 0.05$, and $N_{m_j} = 25000$.

References

[1] Keeling MJ, Rohani P (2008) Modeling infectious diseases in humans and animals. Princeton: Princeton University Press. URL http://www.loc.gov/catdir/toc/fy0805/2006939548.html.

[2] Diekmann O, Heesterbeek JAP, Roberts MG (2009) The construction of next-generation matrices for compartmental epidemic models. J R Soc Interface .

[3] Chaves LF, Harrington LC, Keogh CL, Nguyen AM, Kitron UD (2010) Blood feeding patterns of mosquitoes: random or structured? Front Zool 7: 3.

[4] Kelly DW, Thompson CE (2000) Epidemiology and optimal foraging: modelling the ideal free distribution of insect vectors. Parasitology 120 (Pt 3): 319-27.

[5] Sota T, Mogi M (1989) Effectiveness of zooprophylaxis in malaria control: a theoretical inquiry, with a model for mosquito populations with two bloodmeal hosts. Med Vet Entomol 3: 337-45.

[6] Killeen GF, McKenzie FE, Foy BD, Bøgh C, Beier JC (2001) The availability of potential hosts as a determinant of feeding behaviours and malaria transmission by african mosquito populations. Trans R Soc Trop Med Hyg 95: 469-76.

[7] Killeen GF, Seyoum A, Knols BGJ (2004) Rationalizing historical successes of malaria control in africa in terms of mosquito resource availability management. Am J Trop Med Hyg 71: 87-93.

[8] Hassanali A, Nedorezov LV, Sadykou AM (2008) Zooprophylactic diversion of mosquitoes from human to alternative hosts: A static simulation model. Ecological Modelling 212: 155–161.

[9] Wonham MJ, Lewis MA, Rencławowicz J, van den Driessche P (2006) Transmission assumptions generate conflicting predictions in host-vector disease models: a case study in west nile virus. Ecol Lett 9: 706-25.

[10] Unnasch RS, Sprenger T, Katholi CR, Cupp EW, Hill GE, et al. (2006) A dynamic transmission model of eastern equine encephalitis virus. Ecol Modell 192: 425-440.

[11] Thomas D, Urena B (2001) A model describing the evolution of west nile-like encephalitis in new york city. Mathematical and Computer Modelling 34: 771–781.

[12] Savage HM, Aggarwal D, Apperson CS, Katholi CR, Gordon E, et al. (2007) Host choice and west nile virus infection rates in blood-fed mosquitoes, including members of the culex pipiens complex, from memphis and shelby county, tennessee, 2002-2003. Vector Borne Zoonotic Dis 7: 365-86.

[13] Liu R, Shuai J, Wu J, Zhu H (2006) Modeling spatial spread of west nile virus and impact of directional dispersal of birds. Math Biosci Eng 3: 145-60.

[14] Hartemink NA, Davis SA, Reiter P, Hubálek Z, Heesterbeek JAP (2007) Importance of bird-to-bird transmission for the establishment of west nile virus. Vector Borne Zoonotic Dis 7: 575-84.

[15] Shaman J (2007) Amplification due to spatial clustering in an individual-based model of mosquito-avian arbovirus transmission. Trans R Soc Trop Med Hyg 101: 469-83.

[16] Kilpatrick AM, Kramer LD, Campbell SR, Alleyne EO, Dobson AP, et al. (2005) West nile virus risk assessment and the bridge vector paradigm. Emerg Infect Dis 11: 425-9.

[17] Kenkre V, Parmenter R, Peixoto L, Sadasiv L (2005) A theoretical framework for the analysis of the west nile virus epidemic. Mathematical and Computer Modelling 42: 313–324.
